# Targeted Nanoparticle Delivery CRISPR/Cas9: overcoming biological barriers, enhancing stability, and improving therapeutic precision

**DOI:** 10.1016/j.ijpx.2026.100643

**Published:** 2026-08-19

**Authors:** Sriwidodo Sriwidodo, Cecep Suhandi, Salsa Sagitasa, Belva Annora Alfita Davinali, Iman Permana Maksum, Sabreena Safuan

**Affiliations:** a Department of Pharmaceutics and Pharmaceutical Technology, Faculty of Pharmacy, Universitas Padjadjaran, Sumedang 45363, Indonesia; b Center of Pharmaceutical Formulation Development, Faculty of Pharmacy, Universitas Padjadjaran, Sumedang 45363, Indonesia; c Central Laboratory, Universitas Padjadjaran, Sumedang 45363, Indonesia; d Departement of Chemistry, Faculty of Mathematics and Natural Sciences, Universitas Padjadjaran, Sumedang 45363, Indonesia; e Department of Biomedicine, School of Health Sciences, Universiti Sains Malaysia, Kubang Kerian 16150, Kelantan, Malaysia

**Keywords:** CRISPR/Cas9, Stimuli-responsive nanoparticle, Surface-modified nanoparticle, Targeted delivery, Gene therapy

## Abstract

Clustered regularly interspaced short palindromic repeats (CRISPR)/CRISPR-associated protein 9 (Cas9) has emerged as a promising gene-editing platform for genetic disorders; however, its in vivo application remains limited by low delivery efficiency and biological barriers. Many CRISPR payloads fail to reach target sites due to extracellular degradation, immune clearance, and intracellular trafficking limitations. This review examines the interplay between biological barriers and nanoparticle engineering strategies for CRISPR/Cas9 delivery. A barrier-oriented engineering approach is proposed as a central framework, encompassing ligand-based surface modification for enhanced targeting and uptake, improved circulation stability via PEGylation and biomimetic coatings, and optimized payload release through endosomal escape strategies. Stimulus-responsive nanoparticle systems further enable spatiotemporal control over payload release. Nuclear targeting strategies, including optimization of nuclear localization signals (NLS) and exploitation of endogenous trafficking pathways, are highlighted as key factors for improving genome-level editing efficiency. Despite these advances, major challenges—including limited intracellular delivery efficiency, insufficient targeting precision, and safety concerns—continue to hinder clinical translation. Future directions highlight artificial intelligence-driven nanoparticle design, personalized delivery systems, and next-generation CRISPR platforms. Overall, an integrated, barrier-oriented engineering strategy is essential for advancing CRISPR/Cas9 delivery toward clinical applications, ultimately advancing global good health and well-being.

## Graphical abstract

**Figure:**
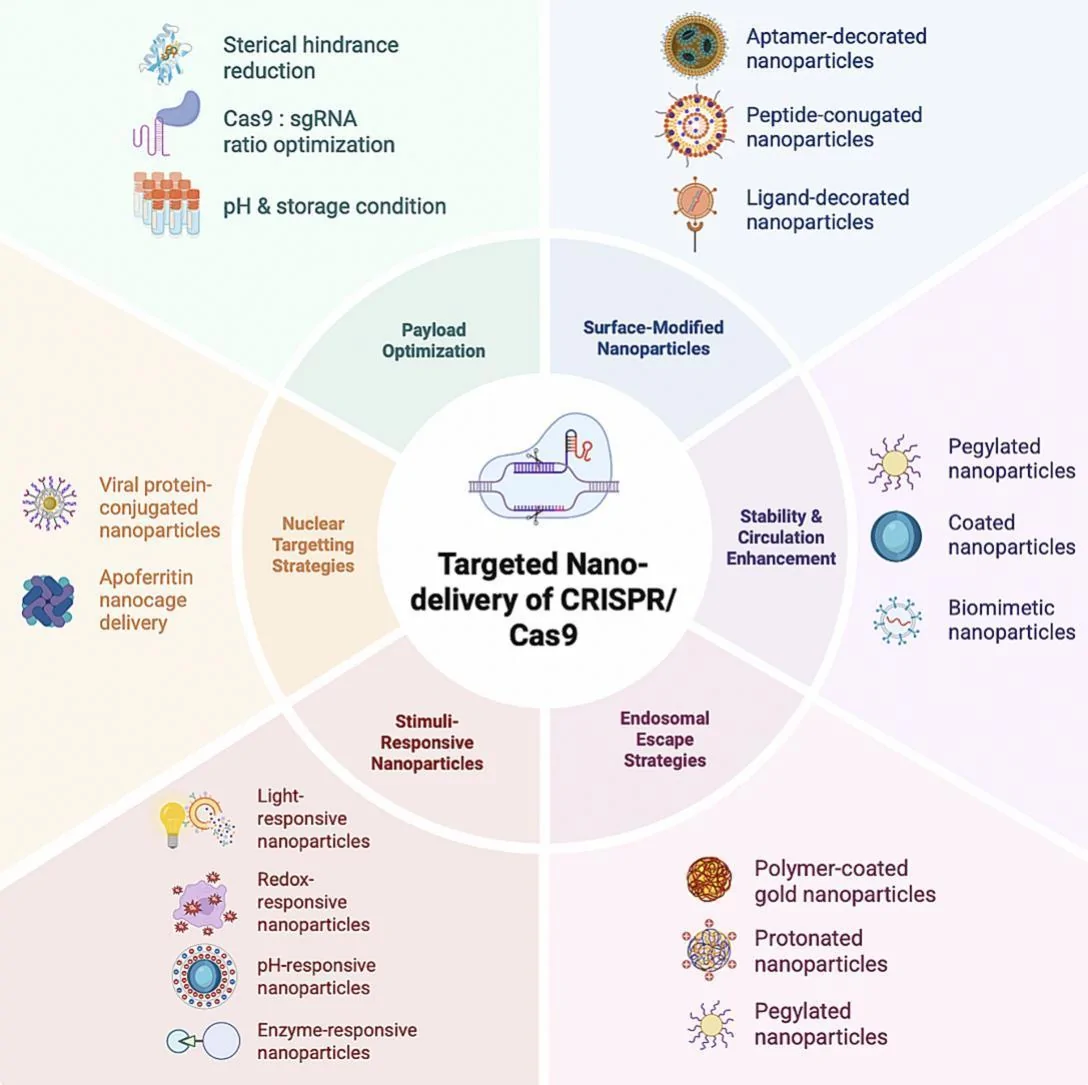

## 1 Introduction

The advancement of gene editing technologies has revolutionized approaches to understanding and treating a wide range of diseases, particularly those with a genetic basis. It is estimated that more than 10,000 human diseases are directly associated with genetic abnormalities, the majority of which still lack effective curative therapies (Gürkan and Satkın, 2025; Shu et al., 2023). Conventional therapeutic approaches are generally symptomatic or target downstream biological pathways, and thus fail to address the root causes of disease (Joshi et al., 2024; Li et al., 2025a). In this context, gene editing technologies offer the potential to directly and precisely modify the genome, thereby enabling more durable therapeutic effects (Ansori et al., 2023; Joo et al., 2025). The global market value of gene editing–based therapies is projected to reach tens of billions of dollars within this decade, reflecting the high level of interest and clinical translational potential of this technology (Wong et al., 2023; Xiang et al., 2026).

Among the various platforms developed, clustered regularly interspaced short palindromic repeats (CRISPR)/CRISPR-associated protein 9 (Cas9) has become the most dominant system due to its design simplicity, high flexibility, and relatively superior efficiency compared with earlier-generation technologies (Kolanu, 2024). This system enables site-specific DNA cleavage with editing efficiencies that can exceed 70–90% under in vitro conditions, depending on cell type and target gene (Lv et al., 2025). In addition, numerous preclinical studies have demonstrated the potential of CRISPR/Cas9 in treating genetic disorders such as sickle cell disease and β-thalassemia, as well as in cancer therapy through engineered immune cells (Butt et al., 2026; Lu et al., 2022; Navaridas et al., 2023). Several CRISPR-based therapeutic candidates have already entered clinical trial stages, marking a critical transition from basic research to clinical application (Kim et al., 2024; Lin et al., 2017; Ottaviano et al., 2022).

However, the success of CRISPR/Cas9 in in vitro systems cannot be directly translated to in vivo settings. One of the major challenges is the efficient delivery of CRISPR components to target tissues (Sioson et al., 2021). Studies indicate that in vivo delivery efficiency often decreases dramatically to below 1–10%, primarily due to nuclease-mediated degradation, rapid elimination by the reticuloendothelial system (RES), and barriers to cellular membrane penetration (Chahal et al., 2026; Haghighi et al., 2024). Furthermore, more than 90% of systemically administered nanoparticles are reported to accumulate in non-target organs such as the liver and spleen, further reducing therapeutic efficiency in desired tissues (Brodin et al., 2025; He et al., 2024). Additional barriers, such as endosomal entrapment, also play a critical role, with estimates suggesting that less than 2% of nanoparticle payloads successfully escape from endosomes into the cytoplasm, thereby significantly limiting gene editing effectiveness (Allen and Yokota, 2024; Zheng et al., 2023).

To address these limitations, nanoparticles have emerged as highly promising delivery systems. Lipid-based nanoparticles (LNPs), for instance, have demonstrated high encapsulation efficiency and the ability to enhance the stability of genetic payloads in circulation (Kim et al., 2023a; Manda et al., 2025). Recent studies report that optimization of LNPs can increase in vivo editing efficiency to over 50% in specific tissues, particularly the liver (Chen et al., 2025; Wang et al., 2025a). In addition, nanoparticle surface engineering through polyethylene glycol (PEG) modification has been shown to prolong circulation half-life and reduce immune clearance. Other approaches, such as pH- or enzyme-responsive nanoparticles, have also demonstrated significant improvements in intracellular payload release, particularly in overcoming endosomal barriers (Albasiony et al., 2025; Wu et al., 2026).

**Fig. 1:**
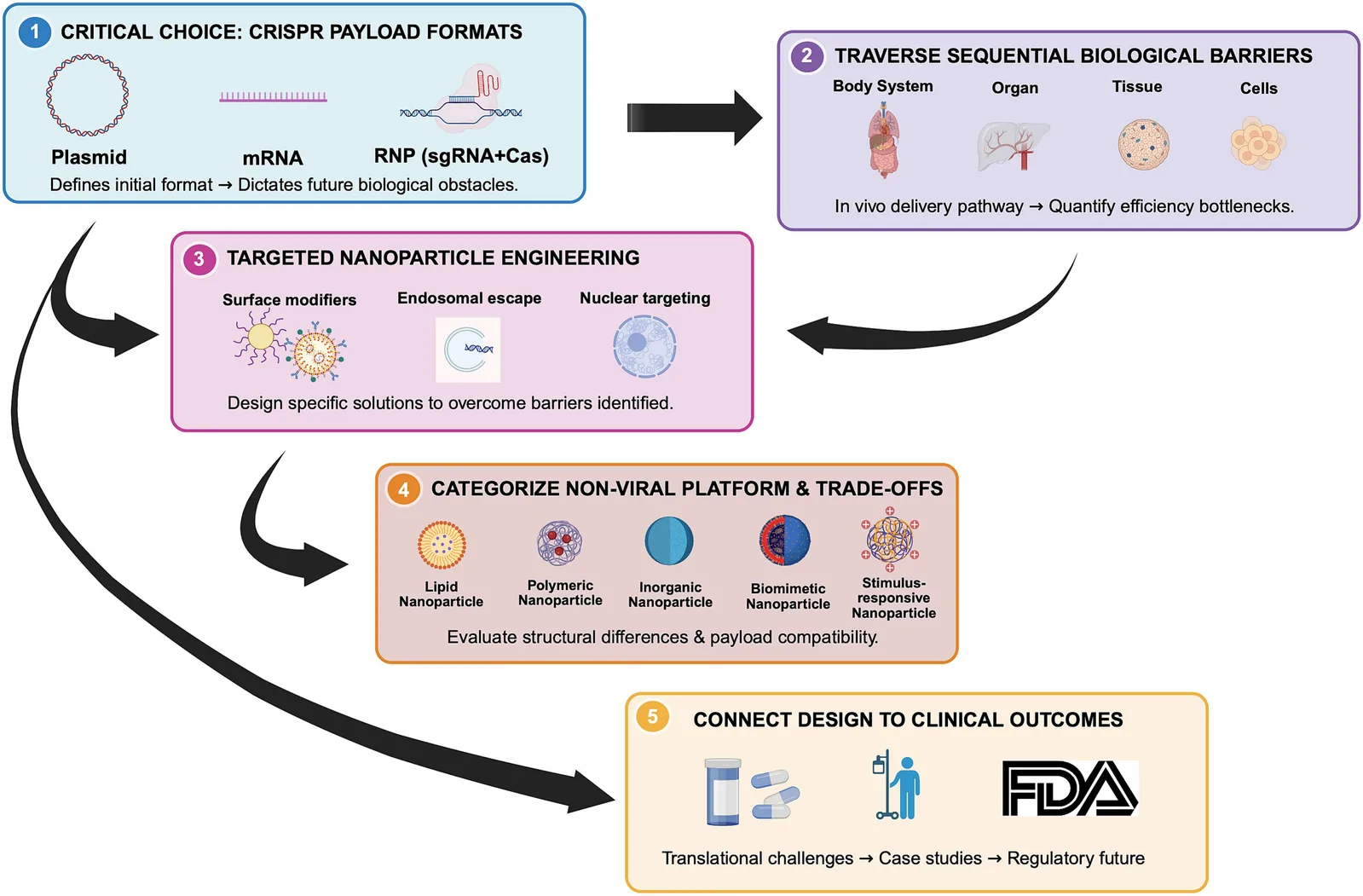
Conceptual framework and progressive narrative pipeline of the review. Schematic diagram illustrating the logical problem-to-solution workflow. Created with BioRender.com.

While several excellent recent reviews have discussed non-viral vectors for CRISPR/Cas9 delivery—focusing on general classifications of stimuli-responsive nanocarriers (Lee et al., 2025), comparisons between physical/viral/non-viral methods with an emphasis on Cas9 protein aggregation (Seijas et al., 2025), or cancer-specific nanoparticle applications (Rauf et al., 2025)—a critical gap remains in systematically connecting nanocarrier design parameters directly to quantitative biological barriers across multiple therapeutic domains. Unlike existing literature, this review establishes a comprehensive, barrier-oriented engineering framework that correlates specific physicochemical modifications (e.g., N/P ratios, formulation pH, and cleavable PEG dynamics) with quantitative delivery efficiency metrics at each sequential biological step—from systemic circulation and protein corona formation to endosomal escape and nuclear transport. Furthermore, this review provides a holistic synthesis of payload optimization strategies (pDNA vs. mRNA vs. RNP stoichiometry and architectural engineering) integrated with hybrid and next-generation CRISPR platforms, offering actionable engineering guidelines for clinical translation beyond a single disease model. By establishing this logical problem-to-solution pipeline, as schematically mapped in Fig. 1, this review aims to guide the rational design of next-generation nanocarriers for clinical genome editing.

## 2 CRISPR/Cas9 therapeutic landscape

The CRISPR/Cas9 system operates through an RNA-guided DNA cleavage mechanism at specific genomic target sites. This complex consists of the Cas9 enzyme as an endonuclease and a single guide RNA (sgRNA) that recognizes the target DNA sequence through complementary base pairing. Once sgRNA directs Cas9 to the specific locus, the enzyme induces a double-strand break (DSB) in the DNA (Xu and Li, 2020). The cell subsequently repairs this damage through two primary pathways: non-homologous end joining (NHEJ), which is error-prone and often results in insertion or deletion mutations, and homology-directed repair (HDR), which enables more precise repair when a homologous DNA template is available. This mechanism underlies various therapeutic applications, including gene knockout, mutation correction, and gene insertion (Liao et al., 2024).

**Fig. 2:**
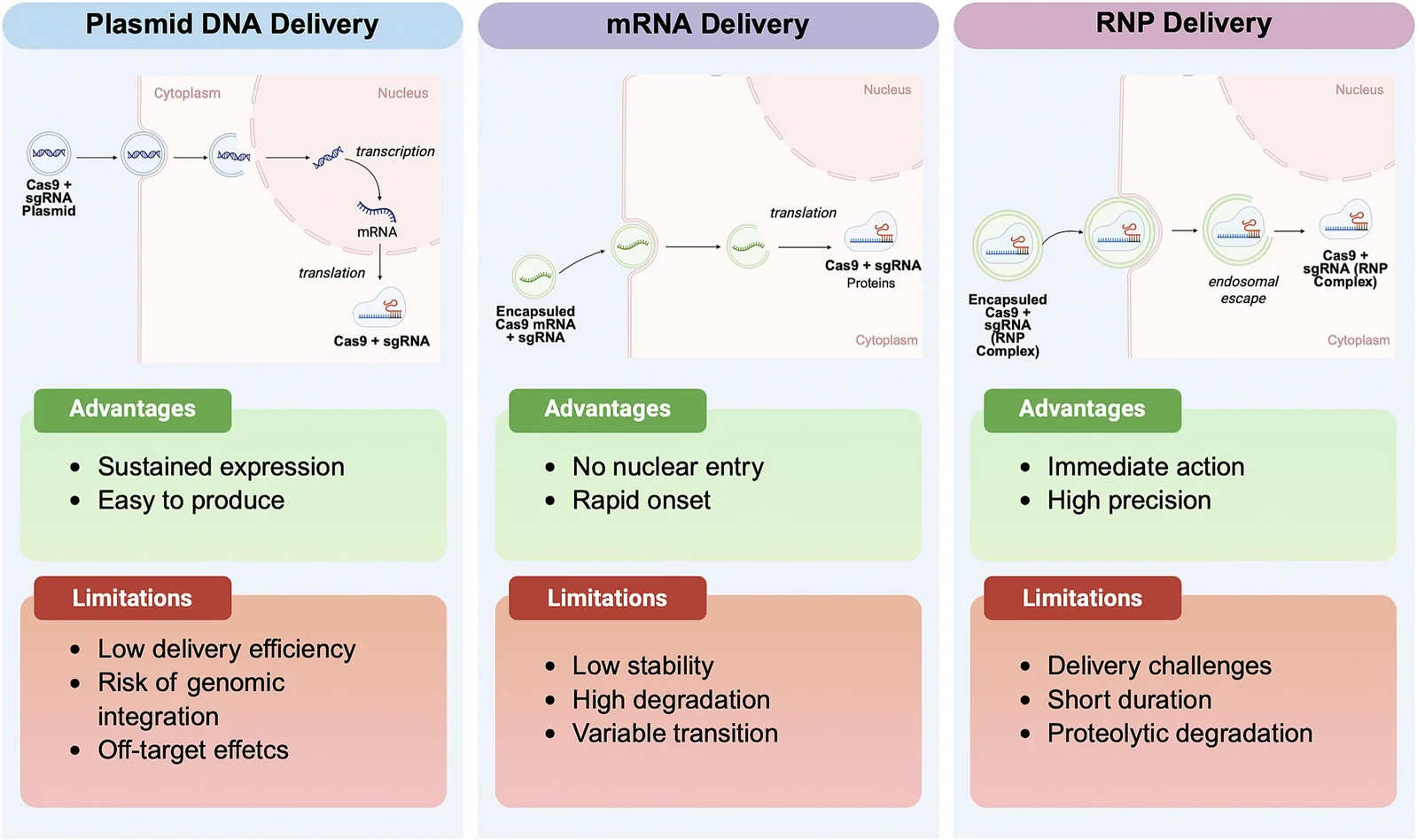
Comparative therapeutic landscape and intracellular mechanisms of CRISPR/Cas9 delivery formats. Schematic comparison of the three primary cargo formats utilized for genome editing: plasmid DNA, mRNA, and ribonucleoprotein (RNP) complexes. (Left) Plasmid DNA Delivery: Following cellular internalization, plasmid DNA must cross the nuclear envelope to undergo transcription into mRNA and subsequent cytoplasmic translation into Cas9 protein and sgRNA. While providing sustained expression and ease of production, it suffers from low delivery efficiency, risk of genomic integration, and elevated off-target effects due to prolonged Cas9 exposure. (Middle) mRNA Delivery: Encapsulated Cas9 mRNA and sgRNA bypass the nuclear entry barrier for protein expression, requiring only cytoplasmic translation. This yields a rapid onset of activity without integration risk, but is limited by low RNA stability, susceptibility to enzymatic degradation, and variable transfection kinetics. (Right) RNP Delivery: Direct delivery of pre-assembled Cas9 protein–sgRNA complexes offers immediate gene editing action, maximum precision, and minimal off-target cleavage owing to its short duration of activity. However, RNP delivery faces severe delivery challenges, including rapid proteolytic degradation and structural vulnerability during intracellular transport. Created with BioRender.com.

In therapeutic applications, CRISPR/Cas9 components can be delivered in several main formats: plasmid DNA (pDNA), messenger RNA (mRNA), and ribonucleoprotein (RNP) (Fig. 2). Each format exhibits distinct physicochemical and biological characteristics that directly influence delivery efficiency, expression duration, and safety profiles (Cheng et al., 2021; Teboul et al., 2020). Therefore, the selection of delivery format is a critical factor in designing CRISPR/Cas9-based therapeutic systems, particularly in relation to biological delivery barriers.

Delivery in the form of pDNA represents one of the earliest approaches due to its relatively high stability and ease of production (Xiu et al., 2023). pDNA contains gene sequences encoding Cas9 and sgRNA, allowing transcription and translation to occur after cellular entry to produce active components (Cheng et al., 2021). The primary advantage of this approach lies in its ability to generate sustained expression, which can enhance the likelihood of gene editing (Song et al., 2021). However, the large size of plasmids limits delivery efficiency, particularly via non-viral nanoparticles (Sioson et al., 2021). Moreover, prolonged expression increases the risk of off-target effects and unintended genomic integration, raising safety concerns for clinical applications (Deng et al., 2021).

As an alternative, delivery in the form of mRNA offers a safer profile, as it does not require nuclear entry for expression (Wang et al., 2025b). mRNA encoding Cas9 can be directly translated in the cytoplasm following cellular uptake, enabling rapid onset of editing activity (Cheng et al., 2021). Its transient nature also reduces the risk of prolonged expression and off-target effects (Teboul et al., 2020). However, mRNA exhibits low stability and is highly susceptible to degradation by ribonucleases, necessitating delivery systems that provide optimal protection (Wang et al., 2025c). Another challenge is the variability in translation efficiency depending on cell type and microenvironmental conditions (Liu et al., 2025; Zheng et al., 2025).

The RNP format, consisting of a direct complex between Cas9 protein and sgRNA, is currently considered the most precise approach in many applications (Cheng et al., 2021). Because it bypasses transcription and translation processes, RNP becomes active immediately upon reaching the cytoplasm or nucleus, resulting in a rapid onset of editing (Albanese et al., 2022). Additionally, its relatively short activity duration significantly reduces off-target risks (Amiri et al., 2025). Nevertheless, the large size and protein nature of the complex, along with its negative charge, present challenges in delivery, particularly in terms of membrane penetration and circulation stability (Sioson et al., 2021). RNP is also more susceptible to proteolytic degradation, requiring specially designed nanoparticle systems to preserve its integrity and bioactivity (Westarp et al., 2025).

Overall, each CRISPR/Cas9 delivery format presents complementary advantages and limitations, and no single approach is universally optimal for all therapeutic applications. The selection of an appropriate format must consider the balance between editing efficiency, safety, and the ability of the delivery system to overcome biological barriers. Therefore, integration between CRISPR/Cas9 payload design and nanoparticle engineering is essential for optimizing the performance of gene editing–based therapies.

## 3 Biological barriers to CRISPR/Cas9 delivery

While selecting the appropriate CRISPR cargo format (pDNA, mRNA, or RNP) establishes the temporal dynamics and specificity of gene editing, the clinical utility of any chosen payload is ultimately constrained by its interaction with host biological systems. Each cargo format imposes distinct physicochemical properties—such as charge density, molecular weight, and enzymatic susceptibility—that dictate how severely it encounters physiological obstacles. Therefore, understanding the sequential biological barriers encountered in vivo represents the crucial next step in rational delivery design.

**Fig. 3:**
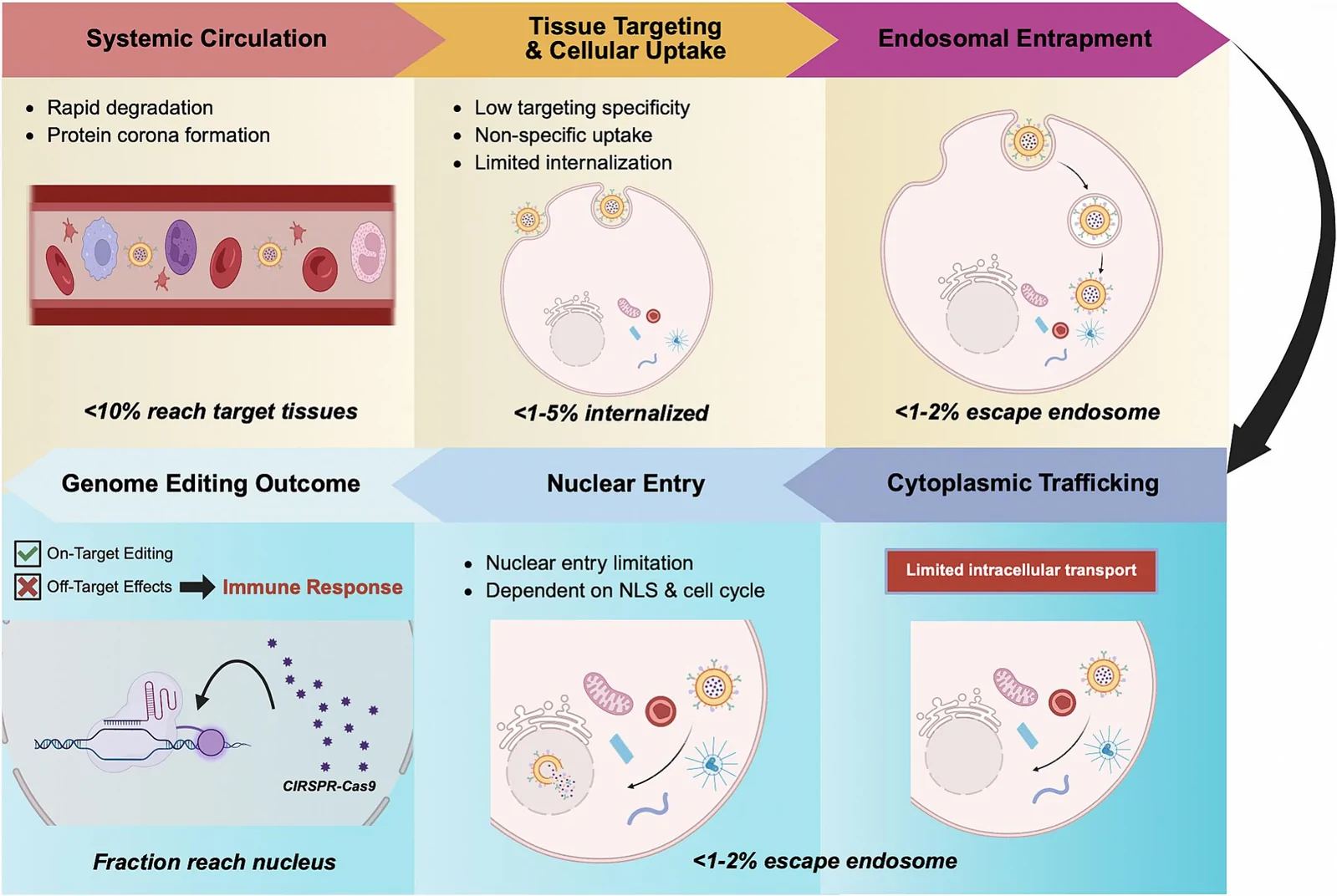
Sequential biological barriers and quantitative efficiency bottlenecks limiting systemic CRISPR/Cas9 delivery. Stepwise illustration depicting the complex physiological obstacles encountered by nanoparticle-encapsulated CRISPR therapeutics from administration to target genome editing. Systemic Circulation: Rapid enzymatic clearance, immune recognition, and non-specific plasma protein adsorption (protein corona formation) severely restrict bio-distribution, leaving <10% of administered dose capable of reaching target tissues. Tissue Targeting & Cellular Uptake: Non-specific biodistribution and receptor heterogeneity result in <1–5% of nanoparticles being internalized into target cells via endocytosis. Endosomal Entrapment: Following internalization, nanoparticles are sequestered within early/late endosomes, where entrapment and subsequent lysosomal degradation allow only <1–2% of cargo to escape into the cytosol. Cytoplasmic Trafficking & Nuclear Entry: Released CRISPR components face restricted intracellular diffusion through the dense cytoskeletal network; subsequently, nuclear entry remains highly dependent on nuclear localization signal (NLS) recognition and host cell cycle status. Genome Editing Outcome: Successful nuclear localization leads to target locus cleavage (on-target editing), but competing off-target cleavage risks can elicit cellular immune responses and cytotoxicity. Created with BioRender.com.

The success of CRISPR/Cas9-based therapies critically depends on the ability of delivery systems to overcome a series of complex and interconnected biological barriers (Fig. 3). These barriers occur sequentially from systemic circulation to nuclear targeting, each significantly reducing gene editing efficiency. In general, in vivo delivery efficiency of CRISPR systems is substantially lower than in vitro conditions, with functional efficiencies often reported below 1–10% in many non-hepatic tissues (Sioson et al., 2021). This highlights delivery as a key determinant in the success of gene editing therapies.

### 3.1 Extracellular barriers

At the initial stage following systemic administration, CRISPR/Cas9 components encounter an extracellular environment that is highly protective against foreign particles. One major barrier is nuclease-mediated degradation, where pDNA and mRNA can undergo rapid degradation in circulation, with reported half-lives ranging from minutes to hours in the absence of adequate protection (Han et al., 2020; Sreekanth et al., 2025). This leads to a significant reduction in the amount of active payload reaching target cells.

In addition, the RES or mononuclear phagocyte system (RES/MPS) plays a critical role in nanoparticle clearance from circulation (Mochalova et al., 2023; Zelepukin et al., 2024). Pharmacokinetic studies indicate that more than 80–90% of intravenously administered nanoparticles accumulate in the liver and spleen within hours post-administration (Yuan et al., 2023). This accumulation is largely mediated by Kupffer cells in the liver, which efficiently capture foreign particles via phagocytosis (Samuelsson et al., 2017). Consequently, only a small fraction of nanoparticles remains available to reach non-hepatic target tissues.

Another significant phenomenon is the formation of a protein corona, referring to the adsorption of plasma proteins such as albumin, immunoglobulins, and fibrinogen onto the nanoparticle surface (Wu et al., 2021). This process occurs within seconds to minutes after nanoparticles enter circulation and can alter hydrodynamic size and surface charge (Sioson et al., 2021). These changes often result in the loss of targeting capability, as surface ligands may be masked by the protein layer. Furthermore, the protein corona can enhance immune recognition, thereby accelerating systemic clearance (Kim et al., 2023b).

### 3.2 Cellular uptake barriers

After surviving in circulation, nanoparticles must effectively interact with target cells and undergo internalization. However, cellular uptake efficiency is often a major limitation, particularly in cells with low endocytic activity. Several studies report that only approximately 1–5% of nanoparticles interacting with target tissues are successfully internalized by target cells (Sioson et al., 2021).

Another critical issue is non-specific uptake, where nanoparticles are taken up by non-target cells such as macrophages, endothelial cells, or cells in other organs (Ernst et al., 2021; Ray et al., 2021). This can lead to undesirable biodistribution and increased risk of off-target toxicity. In many cases, non-specific accumulation in the liver can exceed 50% of the administered dose, highlighting the low targeting efficiency in other tissues (He et al., 2024). Therefore, the ability of nanoparticles to selectively recognize and bind target cells is crucial for improving therapeutic outcomes.

### 3.3 Endosomal escape

Following internalization via endocytosis, nanoparticles are typically entrapped within endosomal vesicles (Hagedorn et al., 2024). This stage represents one of the most significant bottlenecks in CRISPR/Cas9 delivery systems. As endosomes mature into lysosomes, they become enriched with hydrolytic enzymes and acidic pH, leading to degradation of genetic payloads and Cas9 protein (Tornero-Écija et al., 2023).

The efficiency of payload release from endosomes into the cytoplasm is extremely low, with studies estimating that less than 1–2% successfully escape (Chahal et al., 2026). This low efficiency constitutes a major limiting factor, as CRISPR components must reach the cytoplasm or nucleus in an active form. Even in systems with high internalization rates, failure in endosomal escape can render nearly all delivered payload biologically inactive.

### 3.4 Intracellular trafficking and nuclear entry

After reaching the cytoplasm, CRISPR/Cas9 systems still face challenges in intracellular trafficking and nuclear transport. This step is particularly critical for DNA-based formats and most RNP applications that require direct access to genomic DNA (Sioson et al., 2021). Passive diffusion of large macromolecules such as Cas9 complexes is highly limited, necessitating efficient active transport mechanisms (Cui et al., 2021).

This challenge is further exacerbated in non-dividing cells, where the nuclear envelope remains intact and restricts entry of large molecules (Bai et al., 2017). Studies show that nuclear transport efficiency is significantly lower under these conditions compared to dividing cells, where transient nuclear envelope disassembly facilitates access (Sakuma et al., 2021; Sylvers et al., 2023). Although nuclear localization signals (NLS) can enhance transport through nuclear pores, the limited capacity of these pores remains a constraint for large macromolecules (Li et al., 2025c). As a result, only a small fraction of CRISPR payload reaches the nuclear target site.

### 3.5 Off-target effects and immune response

Beyond physical and biological barriers, safety considerations remain a major concern in CRISPR/Cas9-based therapies. One key issue is off-target effects, where DNA cleavage occurs at unintended genomic sites due to imperfect sgRNA recognition (Kalter et al., 2025). Genomic studies indicate that off-target frequencies vary depending on sgRNA design, with some cases exceeding 10% of total editing events under certain conditions (Doench et al., 2016).

In addition, the bacterial origin of Cas9 protein can trigger immune responses (Stigzelius et al., 2025). Reports have identified pre-existing antibodies and T cells against Cas9 in human populations, which may accelerate clearance of CRISPR systems following administration (Hakim et al., 2021). Innate immune activation may also occur through recognition of foreign RNA by receptors such as Toll-like receptors, leading to inflammatory responses (Esparza-Garrido and Velázquez-Flores, 2025). These immune reactions not only reduce therapeutic efficiency but also increase the risk of adverse effects in clinical applications.

Overall, biological barriers in CRISPR/Cas9 delivery are complex, quantitatively significant, and highly interconnected. Understanding these sequential biological barriers—from systemic circulation and cell-specific uptake to endosomal entrapment and nuclear entry—highlights the limitations of passive delivery. To systematically overcome each quantitative efficiency bottleneck, advanced nanoparticle engineering must move beyond simple material selection toward a 'barrier-oriented engineering' framework designed to respond directly to specific physiological obstacles.

## 4 Engineering targeted nanoparticles to overcome barriers

Overcoming the complex biological barriers associated with CRISPR/Cas9 delivery requires nanoparticle engineering approaches that extend beyond material selection, emphasizing instead functional strategies specifically designed to address each stage of these barriers. This “barrier-oriented engineering” paradigm enables the integration of multiple functionalities within a single nanoparticle system, thereby significantly enhancing delivery efficiency. In recent years, a wide range of strategies have been developed to improve structural stability, targeting specificity, endosomal escape, nuclear import, and payload release.

### 4.1 Engineering circulation stability and extracellular stealth

Nanoparticle stability in circulation is a critical determinant of bioavailability and CRISPR/Cas9 system efficacy, particularly in protecting payloads from enzymatic degradation and ensuring optimal distribution to target tissues. One of the most widely employed strategies is PEGylation, which consistently enhances colloidal stability and prolongs circulation time by forming a hydrophilic layer that reduces interactions with plasma proteins and the immune system.

For example, lipidoid-based LNPs modified with DSPE-PEG2k exhibit high stability, with minimal size changes (<25% over 48 hours) and maintained structural integrity for up to 7 days. PEGylation also contributes to low hemolysis (<4%) and predominant liver biodistribution following intravenous administration, supporting efficient RNP delivery with gene knockout levels of up to approximately 70% (Li et al., 2019a). Similarly, core–shell lipid–polymer hybrid nanoparticles based on PEG-PLGA demonstrate remarkable serum stability (≥288 hours) without significant size changes, along with protection against DNase degradation and controlled payload release (>80% over 288 hours). This stability correlates with high gene editing efficiency both in vitro and in vivo, as well as significant therapeutic effects in leukemia models (Liu et al., 2018).

However, data in Table 1 also indicate that PEGylation alone does not always yield optimal performance. In alginate-based layered nanoparticles (CS/GA/PEG), PEG induces burst release (approximately 85%), reducing payload protection and editing efficiency (Alallam et al., 2021). In contrast, chitosan-based coatings provide stronger protection with very slow release (<4%), resulting in higher editing efficiency (approximately 18–36%) (Chang et al., 2026). These findings underscore the importance of balancing stability and payload release in nanoparticle design.

**Table 1:** Stability and circulation strategies in CRISPR/Cas9 nanoparticle systems.

Nanoparticle System	Stabilization Strategy	Key Components	CRISPR Cargo	Stability & Circulation Parameters	Study Model	Key Findings⁎)	Ref.
Lipidoid-based LNP (O16B/N16B tails + helper lipids)	PEGylation (DSPE-PEG2k) for colloidal stability and reduced aggregation; electrostatic self-assembly stabilizes RNP; bilayer vesicle protects cargo	Lipidoid (O16B/N16B), cholesterol, DOPE, DSPE-PEG2k	Cas9:sgRNA RNP complex	Size 100–350 nm; PDI 0.1–0.3; high loading (95–97%); stable ≥48 h (Δsize <25%); structural integrity maintained (FRET <±5%/3 d; <±12.5%/7 d); low hemolysis (<4%); liver-dominant biodistribution (i.v.)	In vitro (HeLa, GFP-HEK) & in vivo (Balb/c mice)	High uptake (∼88%); efficient knockout (∼70%); some formulations outperform Lipofectamine; low cytotoxicity (>80% viability); strong liver accumulation → systemic delivery potential	(Li et al., 2019a)
Core–shell lipid–polymer hybrid NP (CLAN, PEG-PLGA)	PEGylation for serum stability; cationic lipid enhances complexation; core–shell protects from DNase and enables controlled release	PEG5K-PLGA11K, BHEM-Chol	CRISPR plasmid (pCas9 ± gRNA)	Size ∼130–180 nm; PDI ∼0.17–0.33; ζ up to +27.6 mV; EE up to 96.4%; stable ≥288 h; release ∼18% (8 h), >80% (288 h); DNase-resistant	In vitro & in vivo (CML models)	High transfection (∼54–75%); indel ∼46.8% (in vitro), ∼16–19% (in vivo); off-target <1%; strong therapeutic effects (↓ WBC, ↑ survival)	(Liu et al., 2018)
pH-sensitive peptide-modified dual nanocarrier (HuR CRISPR/omSLN-HAR + DTX/omLip-HAR)	pH-cleavable omPEG (stealth → de-coating in TME); multifunctional peptide (targeting, penetration, nuclear localization)	SLN, liposome, DSPE-omPEG, H/A/R peptide	CRISPR plasmid + DTX	Size <200 nm; PDI 0.14–0.21; EE >90%; stable ≥24 weeks; pH-triggered release (>90%/72 h, pH 6.0)	In vitro & in vivo (TNBC model)	Tumor-specific delivery; high transfection (∼52%); synergistic chemo–gene therapy; ↓ tumor growth (∼26% residual); low systemic toxicity	(Lo et al., 2024)
PBAE/pDNA NPs	Electrostatic polyplex formation → DNA condensation & protection	PBAE polymer, plasmid DNA	Cas9 plasmid / shRNA	Size ∼110–174 nm; ζ +13–22 mV; optimal release ratio 60:1; limited systemic stability	In vitro & in vivo (HPV model)	Improved transfection; ↓ HPV E7; tumor inhibition; phenotype reversal	(Zhu et al., 2018)
Chitosan–sgRNA NPs with LbL-coated IOL	Electrostatic crosslinking + multilayer LbL coating for sustained release	Chitosan, PEI, heparin	CRISPR plasmid + sgRNA	Size ∼100 nm; multilayer ζ variation; release ∼30% (24 h), ∼80% (10 d); DNase protection	In vitro (lens epithelial cells)	Sustained release; mTOR knockout; ↓ proliferation, EMT; low toxicity (>70% viability)	(Chang et al., 2026)
Lipid-coated MSN (RNP-LCMSN)	Lipid coating + PEGylation; electrostatic interaction stabilizes RNP	MSN, DOTAP, DOPE, cholesterol, DSPE-PEG2000	CRISPR RNP	Size ∼250–300 nm; PDI ≤0.2; stable >1 week; ∼94% cargo retention; enzyme protection	In vitro & in vivo	Editing ∼20%; ∼5× higher than liposomes; ↓ viral infection (∼56.9%); low toxicity	(LaBauve et al., 2023)
Alginate-based coated NPs (CS/GA/PEG)	Ionotropic gelation + polymer coating (CS, GA, PEG)	Alginate, CaCl₂, chitosan, gum arabic, PEG	CRISPR plasmid	Size 218–552 nm; EE ∼90–99%; release: CS slow (<4%), GA moderate (∼40%), PEG burst (∼85%)	In vitro	CS gives best protection & editing (∼18–36%); PEG shows poor performance due to burst release	(Alallam et al., 2021)
CRISPR@LC-MSN	Large-pore encapsulation + lipid coating for charge neutralization and stability	MSN, DOTAP, cholesterol, DOPE, DSPE-PEG	CRISPR RNP & plasmid	Size ∼130–235 nm; PDI low; stable ≥2 years; ∼70% release (72 h)	In vitro & in vivo	Effective RNP delivery (∼10% editing); plasmid less efficient; performance depends on cargo size/charge	(Noureddine et al., 2020)
GS-pDNA-CMs (RBCM/HCM-coated)	Biomimetic membrane coating → immune evasion, charge shielding	Gemini surfactant, pDNA, RBCM/HCM	Cas9 plasmid + sgRNA	Size <300 nm; PDI <0.3; stable ≥24 h; reduced aggregation; prolonged circulation	In vitro & in vivo	Enhanced liver targeting; improved stability & anti-HBV efficacy	(Wu et al., 2024)
UCNPs-Cas9@CM	Membrane coating + photocleavable linker (NIR-triggered release)	UCNPs, avidin, PCB linker, CM	Cas9 RNP	Size ∼64 nm core; stable ≥7 days; reduced macrophage uptake; liver accumulation	In vitro & in vivo	Spatiotemporal CRISPR control; ↓ HBV markers; low toxicity	(Wang et al., 2022)
Biomimetic mineralized CaP NPs (Bm-RNPs)	CaP mineralization for rapid encapsulation; pH-responsive dissolution	CaP, PAA, Cas9 RNP	CRISPR RNP	Size ∼100–200 nm; ∼100% loading; stable physiologically; dissolves at pH <6.5	In vitro	Efficient delivery; ∼20% indel; high specificity; DNA-free editing platform	(Li et al., 2019b)

⁎ **)Notes:** ↓ indicates decrease; while ↑ indicates increase.

Alternative stabilization strategies have also advanced significantly. Biomimetic membrane coatings, such as RBCM/HCM-coated nanoparticles (GS-pDNA-CMs) and UCNPs-Cas9@CM, exhibit strong immune evasion through self-recognition mechanisms that mimic endogenous cells. These systems enhance serum stability (≥24 hours), reduce aggregation, and improve targeting specificity, including up to fourfold increased uptake in hepatocytes. Membrane coatings also reduce macrophage-mediated phagocytosis and prolong circulation time, thereby improving in vivo delivery efficiency (Wang et al., 2022; Wu et al., 2024).

Structural and material-based strategies further contribute to stability. Lipid-coated mesoporous silica nanoparticles (MSNs) and their variants combine silica pore encapsulation with lipid coating to provide dual protection for CRISPR payloads. These systems demonstrate high stability (over one week without aggregation and up to ≥2 years under certain conditions), resistance to enzymatic degradation, and sustained release (approximately 70% over 72 hours), leading to improved editing efficiency compared to conventional liposomes (LaBauve et al., 2023; Noureddine et al., 2020). Similarly, biomimetic mineralization approaches using calcium phosphate enable nearly 100% encapsulation efficiency, strong protection against RNase/DNase, and rapid release under acidic endosomal conditions, supporting efficient RNP delivery with preserved bioactivity (Li et al., 2019b).

Electrostatic interaction-based stabilization remains relevant, particularly in systems such as PBAE/pDNA polyplexes and chitosan-based nanoparticles, which utilize DNA condensation to enhance protection and cellular uptake. However, these systems often exhibit limited systemic circulation stability, making them more suitable for local delivery or specific administration routes (Zhu et al., 2018).

Innovative strategies such as pH-cleavable PEG (omPEG) in dual nanocarrier systems demonstrate dynamic optimization of stability and circulation. In this approach, PEG functions as a stealth layer during circulation but detaches under acidic tumor microenvironment conditions, thereby enhancing cellular uptake and selective payload release. This strategy effectively addresses the classical trade-off between circulation stability and internalization efficiency (Lo et al., 2024).

### 4.2 Targeted delivery and active tissue tropism strategies

Nanoparticle surface engineering represents a key strategy for enhancing the specificity of CRISPR delivery to target cells through ligand–receptor interactions that facilitate receptor-mediated endocytosis. This approach is significantly more selective than non-specific uptake mechanisms and plays a critical role in improving internalization efficiency, nuclear accumulation, and genome editing effectiveness.

Various systems summarized in Table 2 demonstrate that aptamer-based ligands are among the most effective strategies. For instance, AS1411 aptamer-modified polymer–lipid hybrid nanoparticles (Apt-NPs) targeting nucleolin significantly enhance nuclear accumulation of Cas9, resulting in increased gene editing efficiency (indel and knockout) and reduced expression of target genes such as GFP and Lcn2. These effects directly contribute to the inhibition of cancer cell proliferation and migration (Xu et al., 2026). Similarly, aptamer-decorated LNPs (LNP G + Apt) targeting EpCAM exhibit enhanced selective internalization in EpCAM+ cells, both in vitro and in vivo, leading to more effective gene knockdown and tumor regression in mouse models (Sarkar et al., 2024).

**Table 2:** Surface-modified targeted nanoparticle systems for CRISPR/Cas9 delivery.

Nanoparticle System	CRISPR-Targeted Gene	Targeting Ligand	Study Type	Disease Target	Key Outcomes⁎)	Ref.
AS1411 aptamer-modified polymer–lipid hybrid nanoparticles (Apt-NPs)	GFP; Lcn2	AS1411 aptamer (nucleolin-targeting)	In vitro	Cancer cells (MDA-MB-231, HCT-116)	Enhanced nuclear accumulation of Cas9 → increased gene editing efficiency (indel & knockout) → greater downregulation of target gene expression → improved therapeutic effects (inhibition of cell proliferation and migration)	(Xu et al., 2026)
Triple-targeting plasmid delivery system (P@TSHKP; peptide–polymer nanocarrier)	c-Met	Aptamer TuTu22 (EGFR), SYL3C-HA (EpCAM), HA (CD44)	In vitro & ex vivo (patient whole blood)	Breast cancer (TNBC; BT-549 & circulating malignant cells)	Multi-ligand targeting enhances receptor-mediated uptake (∼10× higher in cancer vs normal cells) → improves CRISPR efficiency (c-Met knockout ∼39.5%) → reduces PD-L1 expression → enhances therapeutic efficacy and anti-tumor immune activation	(Liao et al., 2026)
Aptamer-decorated lipid nanoparticles (LNP G + Apt) for CRISPR plasmid delivery	EpCAM	EpCAM-specific aptamer	In vitro & in vivo (EpCAM+ cells; 4T1 tumor model in mice)	EpCAM-overexpressing cancers (e.g., breast and solid tumors)	Aptamer conjugation improves targeting specificity to EpCAM+ cells → enhances selective internalization vs non-targeted systems → high selectivity (low uptake in EpCAM− cells) → improved plasmid delivery → more effective EpCAM knockdown → inhibition of cancer cell migration and tumor regression in vivo	(Sarkar et al., 2024)
Peptide-based nanoparticles (ADGN-AVA + ADGN-PEG) for CRISPR RNA delivery	Luciferase (model gene)	YIGSR peptide (laminin receptor-targeting)	In vitro & in vivo (A549, MCF7, orthotopic lung tumor model)	Cancers with laminin receptor overexpression	YIGSR ligand enables specific targeting → increases selective uptake in tumor cells (2–5× higher than normal cells) → improves CRISPR RNA delivery → ∼50–60% gene knockdown and ∼60% DNA cleavage → targeted in vivo distribution (notably to lung) → enhanced therapeutic efficacy with minimal toxicity	(Guzman Gonzalez et al., 2024)
Chitosan/HA-LHRH dual-targeted nanoparticles (CS/HA-LHRH/pCRISPR polyplex)	ERCC1	Hyaluronic acid (CD44) + LHRH peptide	In vitro (MCF-7/DDP cells)	Cisplatin-resistant breast cancer	Dual targeting enhances uptake and transfection (∼56% EGFP+ at 48 h, comparable to Lipofectamine with lower toxicity) → protects plasmid from DNase degradation → stable under serum-like conditions → ERCC1 knockdown (∼10 to ∼0.4) → significantly reduces drug resistance (IC50 decrease: ∼99.96 → 9.32 μg/mL at 48 h; 45.5 → 7.22 μg/mL at 72 h) → restores chemosensitivity with high efficiency and low toxicity	(Tavazohi et al., 2023)
Mannose-functionalized hyaluronic acid–poly(ethylene imine) nanoparticles (HA-PEI-Man NPs)	Rosa26 locus; GFP	Mannose (CD206-targeting on hepatocytes and macrophages)	In vitro & in vivo (AML12 hepatocytes; C3He mice)	Liver targeting (e.g., Wilson’s disease relevance)	High liver uptake (∼58–69% hepatocytes) via CD206 targeting → in vivo editing up to ∼15% → enhanced editing with mannose (∼4.7% vs 2.8%) → GFP expression up to ∼17.2% → altered biodistribution (↑ liver & spleen, ↓ kidney) → effective co-delivery of Cas9 and therapeutic payload	(Francis et al., 2022)
PBA-BADP lipid nanoparticles (boronic acid-modified cationic lipid NP)	GFP; HPV18E6	Phenylboronic acid (targets sialic acid on cancer cells)	In vitro (HeLa, HEK293, SiHa, DU145)	Cancer (especially sialic acid-overexpressing cells, e.g., cervical cancer)	PBA–sialic acid interaction enhances selective uptake (∼90% vs ∼10%) → GFP knockout ∼50% → indel efficiency ∼14% (vs ∼8% control) → HPV18E6 editing ∼18.7% → ∼50% reduction in cancer cell viability → high selectivity (minimal effect on normal cells) → mRNA expression increase up to ∼110× → lower cytotoxicity vs non-PBA lipids	(Tang et al., 2019)

⁎ **)Notes:** ↓ indicates decrease; while ↑ indicates increase.

Multi-ligand approaches demonstrate superior performance compared to single-ligand systems. The triple-targeting system P@TSHKP, which combines aptamers against EGFR, EpCAM, and CD44, achieves approximately a tenfold increase in uptake in cancer cells compared to normal cells. This enhancement directly translates into improved CRISPR efficiency, with c-Met gene knockout reaching approximately 39.5% (Liao et al., 2026). Additionally, this system exhibits immunotherapeutic effects through downregulation of PD-L1 expression, potentially enhancing anti-tumor immune responses (Liao et al., 2026). These findings highlight that multi-targeting strategies not only improve specificity but also enable synergistic integration with immunotherapy.

Beyond aptamers, peptide-based ligands also demonstrate strong targeting performance. Nanoparticles functionalized with the YIGSR peptide targeting laminin receptors achieve a 2–5-fold increase in selective uptake in tumor cells and DNA editing efficiencies of up to approximately 60%. In vivo studies further demonstrate more directed biodistribution, such as preferential accumulation in lung tissue in orthotopic models, with minimal toxicity, reinforcing their relevance for therapeutic applications (Guzman Gonzalez et al., 2024).

Combinatorial approaches such as dual-targeting offer additional advantages in addressing receptor heterogeneity. Chitosan/HA-LHRH dual-targeted nanoparticles targeting CD44 and LHRH receptors show significant improvements in transfection efficiency (up to approximately 56% positive cells) and successful editing of the ERCC1 gene, leading to reduced resistance to cisplatin. The substantial decrease in IC50 values indicates that this strategy not only enhances delivery but also provides strong therapeutic implications in overcoming chemoresistance (Tavazohi et al., 2023).

Small ligand-based approaches, including carbohydrates and chemical moieties, also demonstrate high potential for tissue-specific targeting. Mannose-functionalized HA-PEI nanoparticles (HA-PEI-Man NPs) exploit interactions with CD206 receptors on hepatocytes and liver macrophages, resulting in high liver accumulation (approximately 58–69%) and in vivo gene editing efficiencies of approximately 15% (Francis et al., 2022). Meanwhile, PBA-modified LNPs targeting sialic acid on cancer cell surfaces exhibit highly selective uptake (approximately 90% in cancer cells versus approximately 10% in normal cells) and efficient editing of endogenous genes such as HPV18E6 (approximately 18.7%), accompanied by significant reductions in cancer cell viability (Tang et al., 2019).

### 4.3 Stimuli-responsive design for enhanced endosomal escape

Endosomal escape represents one of the most critical steps in CRISPR/Cas9 delivery, as most nanoparticles internalized via endocytosis become trapped within endosomes and are susceptible to lysosomal degradation. Consequently, various material design strategies and stimuli-responsive mechanisms have been developed to enhance cytoplasmic payload release and achieve spatiotemporal control over CRISPR–Cas9 release, as summarized in Table 3 and Table 4.

**Table 3:** Endosomal escape strategies in CRISPR/Cas9 nanoparticle delivery systems.

Endosomal Escape Strategy	Nanoparticle System	CRISPR–Cas9 Target Gene	Escape Mechanism	Study Type	Target Disease	Effects on CRISPR–Cas9 Delivery & Pharmacological Outcomes	Ref.
Polymer-mediated endosomal disruption (PAsp(DET)) + caveolae-mediated endocytosis	CRISPR-Gold (AuNP–DNA–Cas9 RNP–silica–PAsp(DET))	BFP→GFP, CXCR4, dystrophin (DMD), Ai9 locus	PAsp(DET) induces membrane disruption, leading to endosomal destabilization; uptake via caveolae/raft pathway avoids lysosomal degradation and enhances cytosolic escape	In vitro & in vivo (HEK293, hES/hiPS, BMDCs, mdx & Ai9 mice)	Duchenne muscular dystrophy (DMD) & reporter models	Increased HDR efficiency (∼11.3% in vitro; ∼5.4% in vivo); co-delivery of Cas9 RNP and donor DNA; restoration of dystrophin expression; reduced muscle fibrosis; improved muscle function (∼2-fold); low toxicity and minimal immunogenicity	(Lee et al., 2017)
Lipid-mediated endosomal escape + bioreducible intracellular release	Bioreducible lipid nanoparticles (e.g., O14B-based LNPs)	EGFP (reporter gene)	Internalization via clathrin-mediated endocytosis; lipid-mediated endosomal escape with minimal lysosomal colocalization; disulfide bonds degraded in reductive cytosol, releasing active protein/RNP	In vitro & in vivo (HeLa, HEK cells, mouse brain model)	Reporter gene model & potential neurological applications	High protein delivery (>80% uptake); enhanced gene recombination (∼80%); efficient genome editing (∼70% KO); effective Cas9 RNP delivery; localized brain targeting; high cell viability (>85%) with low toxicity	(Wang et al., 2016)
pH-sensitive lipid-mediated escape (DOPE) + Chol-PEG domain optimization	PEGylated cationic liposomes (DOTAP/DOPE/Cholesterol/Chol-PEG lipoplexes)	EGFP	DOPE forms inverted hexagonal phase at acidic endosomal pH, promoting membrane fusion and cytosolic DNA release; Chol-PEG domain distribution enhances uptake and reduces PEG-related trafficking barriers; low pH destabilizes lipoplex for payload release	In vitro (HEK293 cells)	Reporter gene model (EGFP disruption)	Increased transfection efficiency (∼1.3× vs Lipofectamine); improved serum stability; enhanced CRISPR knockout (∼39% EGFP reduction); improved endosomal escape; low cytotoxicity (∼90% viability)	(Hosseini et al., 2019)
pH-switchable zwitterionic lipid + membrane fusion-induced escape	iPhos lipid nanoparticles (iPLNPs; e.g., 9A1P9–5A2-SC8, 9A1P9-DDAB)	Reporter: tdTomato (Ai9), EGFP/Fluc; Endogenous: PTEN	Protonation in acidic endosomes induces zwitterionic state and cone-shaped lipid conformation; triggers lamellar-to-hexagonal phase transition, enhancing membrane fusion and endosomal disruption; followed by nanoparticle disassembly and cargo release	In vitro & in vivo (IGROV-1 cells, C57BL/6 & Ai9 mice)	Reporter models & multi-organ genetic targets	Enhanced mRNA and CRISPR delivery; increased endosomal escape (high hemolysis/fusion at acidic pH); high organ-specific editing (liver, lung); high transfection (∼91% hepatocytes); rapid protein expression (peak ∼6 h); enables co-delivery; low toxicity and repeat dosing capability	(Liu et al., 2021)
pH-responsive imidazole-based escape (protonation-triggered)	ZIF-8 (CC-ZIFs; metal–organic framework)	EGFP	Imidazole protonation at endosomal pH (5–6) induces membrane destabilization and framework degradation, enabling rapid cytosolic release of Cas9/sgRNA and nuclear translocation	In vitro (CHO cells)	Reporter gene model	Rapid nuclear delivery (≤6 h); indel ∼30%; gene knockdown ∼31–37%; significantly higher efficiency than free RNP; improved delivery and editing performance	(Alsaiari et al., 2018)

**Table 4:** Stimuli-responsive nanoparticle systems for CRISPR/Cas9 delivery.

Nanoparticle System	CRISPR Cargo	Stimulus Type	Response Mechanism	Study Type	Disease Target/Model	Key Outcomes⁎)	Ref.
Disulfide cross-linked angiopep-2-functionalized nanoparticles (ANPSS(Cas9/sgGDF15))	Cas9 protein + sgRNA targeting GDF15	Redox (elevated intracellular/TME GSH)	Disulfide bond reduction triggers nanoparticle degradation and intracellular release; angiopep-2 mediates LRP-1 targeting for BBB penetration	In vitro & in vivo (BBB model, orthotopic & spontaneous GBM mice)	Glioblastoma	Stimuli-responsive release enhances specificity and cargo protection → high editing efficiency (∼67% indel) → GDF15 downregulation → TME remodeling (↑ CD8^+^ T cells, ↑ M1, ↓ M2 macrophages) → tumor inhibition, improved survival, synergy with anti-PD-1	(Zou et al., 2025)
GSH-responsive silica nanoparticles (SNP-PEG / SNP-PEG-GalNAc / SNP-PEG-ATRA)	DNA, mRNA, Cas9 RNP, RNP + ssODN	Redox (intracellular GSH)	Disulfide cleavage (BTPD) degrades silica matrix; imidazole enhances proton sponge escape; PEGylation improves stability; ligands enable targeting	In vitro & in vivo (HEK293, Ai14 mice; subretinal & i.v.)	Retina, liver, reporter models	High loading (>90%) and stability; superior transfection vs Lipofectamine; high editing (∼72% indel); HDR capability; enhanced liver targeting (∼2× with GalNAc); excellent biocompatibility	(Wang et al., 2021)
NIR-responsive UCNPs-Cas9@CM	Cas9 RNP targeting HBV cccDNA	Near-infrared (808 nm)	UCNP converts NIR→UV to cleave photocleavable linker → controlled release; biomimetic membrane enables immune evasion & homotypic targeting	In vitro & in vivo (HBV cell lines & transgenic mice)	Hepatitis B	Spatiotemporal-controlled release → significant reduction of HBV DNA, RNA, cccDNA, HBsAg/HBeAg; ∼4× liver targeting; strong immune evasion; low toxicity	(Wang et al., 2022)
Photosensitive cationic liposomes (verteporfin-loaded)	Cas9 RNP	Light (LED 690 nm)	Light activates photosensitizer → singlet oxygen generation → lipid oxidation → endosomal disruption and release	In vitro & in vivo (zebrafish reporter model)	Reporter & inflammation-related genes	High KO efficiency (∼52–53% in vitro; ∼77% in vivo); precise spatial–temporal control; minimal leakage (<3%); low toxicity; reduced off-target	(Aksoy et al., 2020)
Lipid–AuNP hybrid nanoparticles (LACP)	Cas9-sgRNA plasmid (Plk-1)	Laser (514 nm; photothermal)	LSPR-induced heat breaks Au–S bonds & destabilizes lipid shell → controlled release and nuclear delivery	In vitro & in vivo (melanoma model)	Melanoma	High editing (↓ Plk-1 ∼65%); ↑ apoptosis (∼25.9%); tumor inhibition (∼85% reduction); strong tumor retention; low toxicity without irradiation	(Wang et al., 2018)
Silica–MOF hybrid nanoparticles (SMOF NPs)	DNA, mRNA, Cas9 RNP, RNP + ssODN	pH (endosomal/lysosomal)	ZIF degradation at acidic pH + proton sponge effect → rapid release and escape; silica enables stability & functionalization	In vitro & in vivo (Ai14 mice, retinal models)	Retina & multi-cell models	High loading (>90%); enhanced uptake (∼3.2×); improved KO (∼1.3×) and HDR (∼1.4×); rapid escape (≤2–4 h); successful in vivo editing; high biocompatibility	Wang et al. (2020a)
ZIF-90 MOF nanoparticles	Cas9 protein (± sgRNA)	ATP (intracellular)	ATP competes with Zn^2+^ → MOF disassembly → cytosolic release; proton sponge enhances escape	In vitro (HeLa)	Cancer (reporter model)	High encapsulation (>90%); high uptake (>90%); efficient release (>90% at 10 mM ATP); KO ∼40%; good biocompatibility	(Yang et al., 2019)
Esterase-responsive polymeric micelles	Cas9 mRNA + sgRNA (PCSK9)	Enzymatic (CES1)	Esterase-mediated degradation releases mRNA/sgRNA → translation → RNP formation → nuclear editing	In vitro & in vivo (HFD mice)	Hyperlipidemia	Effective PCSK9 editing; ↓ LDL-C (∼20%); liver-specific accumulation; improved metabolic profile; no toxicity	(Zhao et al., 2023)
PEI-coated magnetic nanoparticles (PEI-MNPs)	CRISPR plasmid	Magnetic field + pH	Magnet enhances cell contact; proton sponge enables escape	In vitro	DNA repair model	Moderate increase in transfection (∼13%); stable protection (>24 h); good biocompatibility	(Rohiwal et al., 2020)
Microbubble–nanoliposome hybrids (MB-NL)	Cas9 RNP (SRD5A2)	Ultrasound	Cavitation induces membrane disruption & sonoporation → enhanced uptake and release	In vitro & in vivo	Androgenic alopecia	High in vivo editing (∼71.6%); ↓ gene expression (∼70%); strong hair regeneration (∼90%); high specificity; minimal toxicity	(Ryu et al., 2020)
Ultrasound-propelled Au nanowire nanomotors	Cas9 RNP	Ultrasound + redox (GSH)	Active propulsion enables direct cytosolic entry; GSH cleaves disulfide bonds → release	In vitro	Melanoma	Extremely high KO (∼80–95%); rapid delivery (minutes); effective at low dose; superior to passive uptake; non-toxic	(Hansen-Bruhn et al., 2018)

⁎ **)Notes:** ↓ indicates decrease; while ↑ indicates increase.

One major approach involves the use of endosomal pH-responsive materials. Lipid-based systems such as DOPE-containing lipoplexes and iPhos LNPs exploit acidic conditions (pH ∼5.0–6.5) to trigger molecular structural changes (Hosseini et al., 2019; Liu et al., 2021). In DOPE-based systems, the formation of an inverted hexagonal phase facilitates membrane fusion and DNA release into the cytoplasm (Hosseini et al., 2019). In iPhos systems, protonation of amine groups generates zwitterionic structures that induce conformational changes into cone-shaped lipids, promoting membrane phase transitions and enhanced endosomal disruption. These membrane fusion mechanisms are highly effective, as demonstrated by high delivery efficiency and organ-specific editing, including transfection efficiencies of up to approximately 91% in hepatocytes (Liu et al., 2021).

In addition to lipid systems, pH-responsive strategies using inorganic materials such as metal–organic frameworks have been developed. In ZIF-8 systems, protonation of imidazole groups at endosomal pH destabilizes the framework and induces particle degradation, resulting in rapid release of Cas9/sgRNA into the cytoplasm. This mechanism enhances endosomal escape efficiency while enabling selective payload release without biomolecule denaturation. Experimentally, this system demonstrates nuclear delivery within ≤6 hours, with editing efficiencies of approximately 30% and gene knockdown of approximately 31–37%, significantly outperforming free RNP (Alsaiari et al., 2018). A similar principle is utilized in silica–metal–organic framework hybrid nanoparticles (SMOF NPs), where the ZIF component degrades under acidic endosomal conditions, triggering rapid payload release while the silica matrix provides structural stability for co-loading Cas9 RNP and donor DNA, achieving high editing efficacy in retinal models (Wang et al., 2020a).

Polymer-based strategies also exhibit high effectiveness, particularly through direct membrane disruption mechanisms. In CRISPR-Gold systems, cationic polymers such as PAsp(DET) act as endosomal-disruptive agents following internalization via caveolae-mediated endocytosis, a pathway that avoids lysosomal degradation. This enables simultaneous delivery of Cas9 RNP and donor DNA, achieving significant HDR efficiencies both in vitro and in vivo, along with therapeutic effects such as restoration of dystrophin expression and improved muscle function in disease models (Lee et al., 2017).

Redox-responsive systems utilize intracellular glutathione (GSH) gradients to trigger endosomal escape and cytosolic release. Bioreducible lipid systems undergo clathrin-mediated endocytosis followed by lipid-mediated endosomal escape, wherein disulfide bonds are cleaved in the reductive cytoplasmic environment, releasing active Cas9 RNP to achieve ∼70% gene knockout (Wang et al., 2016). Disulfide cross-linked angiopep-2-functionalized nanoparticles exploit GSH reduction for Cas9 release while enabling blood–brain barrier penetration to achieve ∼67% editing in glioblastoma models (Zou et al., 2025). Similarly, GSH-responsive silica nanoparticles (SNPs) combine disulfide reduction with imidazole-mediated proton sponge effects, yielding up to ∼72% HDR-mediated gene correction (Wang et al., 2021). Furthermore, metabolic triggers such as ATP are leveraged in ZIF-90 systems, where intracellular ATP competitively binds Zn2+ ions, inducing framework disintegration and achieving >90% payload release and ∼40% gene knockout (Yang et al., 2019).

Beyond internal triggers, various external stimuli have been employed to enhance control over CRISPR release. Light-responsive upconversion nanoparticles (UCNPs) convert near-infrared (NIR) light into UV irradiation to cleave photocleavable linkers for spatiotemporal Cas9 release, suppressing HBV replication (Wang et al., 2022). Photothermal strategies using lipid–gold hybrid systems (LACP) utilize laser-induced surface plasmon resonance to generate heat, promoting endosomal membrane penetration and achieving up to ∼65% target protein reduction (Wang et al., 2018). Photosensitive liposomes generating reactive oxygen species (ROS) disrupt endosomal membranes to reach up to ∼77% knockout in vivo (Aksoy et al., 2020). Additionally, ultrasound-based microbubble–nanoliposome complexes leverage acoustic cavitation to transiently permeabilize cell membranes (∼71.6% editing efficiency) (Ryu et al., 2020), while ultrasound-propelled gold nanowire nanomotors actively penetrate cell membranes to achieve ∼80% knockout within 2 hours (Hansen-Bruhn et al., 2018). Enzymatic triggers, such as esterase-responsive micelles, enable hepatocyte-specific Cas9 release via carboxylesterase degradation for PCSK9 editing (Zhao et al., 2023), whereas magnetic guidance (magnetofection) enhances localized cellular uptake (Rohiwal et al., 2020).

Complementary approaches from gene and vaccine delivery fields offer further potential. PLGA nanoparticles with gas-generating agents (ammonium bicarbonate) produce CO_2_ and NH_3_ in acidic endosomes, generating internal pressure that ruptures endosomal membranes (Liu et al., 2015). Polycation–melittin conjugates modified with pH-labile moieties mask membrane-lytic activity at physiological pH while reactivating in acidic endosomes to minimize toxicity (Meyer et al., 2007). Dynamic polyconjugate systems reversibly modify membrane-active polymers, activating them exclusively within endosomal environments for safe in vivo delivery (Rozema et al., 2007). Conversely, while cell-penetrating peptides (CPPs) enhance cellular uptake, recent evidence indicates that increased cytosolic accumulation stems primarily from membrane association rather than superior endosomal escape, highlighting the need to prioritize true endosomal disruption mechanisms (Teo et al., 2021).

### 4.4 Nuclear localizing and intracellular trafficking engineering

To achieve maximal efficacy, CRISPR/Cas9 components must successfully reach the nucleus, particularly in genome DNA editing applications. One of the primary approaches involves the use of NLS, peptide sequences recognized by the cellular nuclear transport machinery. Recent studies indicate that not only the presence of NLS, but also its distribution and density on the Cas9 structure, critically influence nuclear delivery. Internal hiNLS engineering strategies, which incorporate multiple NLS motifs into the Cas9 backbone, have been shown to enhance interactions with importin, thereby strengthening nuclear import and retention, and even enabling nuclear re-import. This approach results in a substantial increase in editing efficiency (up to >80%), although it may also be associated with an elevated risk of off-target effects (Noel et al., 2025).

Beyond NLS-based strategies, alternative and more innovative approaches have emerged to overcome limitations in nuclear transport. One such strategy involves NLS-independent nuclear delivery using stimulus-responsive systems. For example, an apoferritin nanocage platform combined with doxorubicin (DOX) exploits cellular stress responses to induce endogenous nuclear translocation. This system not only facilitates endocytosis and endosomal escape but also enables efficient delivery of Cas9/sgRNA complexes into the nucleus without requiring NLS, achieving considerable editing efficiency in both in vitro and in vivo models. These findings highlight that activation of intrinsic cellular pathways may serve as an effective alternative to direct modification of Cas9 (Sun et al., 2025).

In parallel, several studies demonstrate that subtle alterations in nuclear localization-related elements can produce significant biological effects. For instance, mutations in the NLS of endogenous proteins such as IL-1α can affect subcellular localization and disrupt gene regulation through structural changes in long non-coding RNA (lncRNA), ultimately leading to loss of gene expression (Daniels et al., 2017). Furthermore, in CRISPR-modified viral models, structural alterations in viral proteins can delay nuclear entry of genetic material, resulting in reduced replication and virulence (Ahmed et al., 2025). These observations underscore that nuclear targeting is not merely a delivery challenge, but is also intricately linked to complex molecular regulatory mechanisms.

### 4.5 Payload optimization and stoichiometric engineering

Payload optimization is a critical aspect in the design of nanoparticle-based CRISPR/Cas9 delivery systems, as payload format, stability, and molecular interactions directly influence editing efficiency and therapeutic safety. pDNA delivery enables prolonged expression but carries risks of genomic integration and off-target effects, whereas mRNA offers safer, transient expression despite its susceptibility to intracellular degradation. In contrast, pre-assembled RNP complexes have demonstrated substantial advantages, including rapid onset of activity and superior temporal control over off-target effects (Akhter et al., 2026; Haldrup et al., 2023; Im et al., 2024). Multiple studies report that RNP delivery achieves high editing efficiencies, such as >85–95% on-target editing with minimal exposure, along with improved delivery consistency compared to DNA-based formats (Haldrup et al., 2023). However, RNP efficacy remains highly dependent on compatibility with the delivery system; for instance, WJ11/Cas9 RNP exhibits negligible effects when delivered via CL4H6 LNPs, highlighting that optimal payload formats still require suitable carriers (Akhter et al., 2026).

Payload optimization encompasses several key dimensions. First, nanoparticle architecture and the positioning of Cas9 play a decisive role. Studies on lentiviral nanoparticles (LVNPs) show that C-terminal fusion of Cas9 to GagPol yields relatively low editing efficiency (∼8–38%), whereas repositioning to the N-terminal Gag combined with a membrane-targeting domain (e.g., pH domain) significantly enhances efficiency to >50% and up to ∼82% under optimal conditions (Haldrup et al., 2023). Furthermore, reducing steric hindrance through advanced architectural design—such as LVNP3.0 lacking reverse transcriptase and integrase—enables packaging of larger payloads, including base editors and prime editors, achieving in vivo editing efficiencies of ∼17–32%, base editing of ∼18–45%, and prime editing of ∼5–6% without inducing DSBs (Haldrup et al., 2023).

Second, payload stoichiometry is a key determinant of delivery and editing efficiency. Optimization of the Cas9:sgRNA ratio within RNP complexes enhances formation of active complexes, yielding editing efficiencies up to ∼45.2% (NGS). In LNP systems, increasing the N/P ratio (lipid:sgRNA) improves complexation and encapsulation, reaching a plateau at ratios ≥11. Additionally, lipid composition optimization—such as increasing the proportion of MC3 relative to DSPC—can enhance editing efficiency (∼51.9%), whereas suboptimal compositions reduce performance (Im et al., 2024). Similarly, stoichiometric tuning of plasmids during LVNP production balances particle yield and cargo loading, achieving high editing efficiencies of >75–98% (Haldrup et al., 2023). Notably, a “less is more” strategy—delivering low-dose RNPs with short activity duration—can maintain high efficiency while minimizing off-target effects.

Payload stabilization during formulation and storage is equally crucial. Formulation conditions significantly affect Cas9 biological activity; an optimal pH of ∼6.0 increases Cas9 loading (56.3%) and sgRNA loading (75.3%), enhances nuclear uptake (∼9-fold), and improves editing efficiency (∼53%). In contrast, highly acidic conditions (pH 4.0) lead to complete denaturation and loss of enzymatic activity, whereas pH ≥5.5 preserves ≥95% Cas9 activity. Additional strategies, such as sgRNA heat treatment, improve monomer homogeneity and encapsulation efficiency, which are critical determinants of in vivo editing success. However, storage stability remains a major challenge, with efficiency reductions of up to −61.9% within 24 hours under certain conditions, underscoring the importance of storage optimization (Im et al., 2024).

Carrier design further dictates payload performance. The selection of appropriate lipids, such as MC3 over ALC-0315 or SM-102, results in substantial differences in editing efficiency (with <5% observed for non-optimal lipids) (Im et al., 2024). In vivo, lipid modifications such as CL4F11_ζ−2—with biodegradable and “flower-like” structural features—enhance payload release and produce tangible biological effects, including reductions in hepatitis B virus (HBV), unlike earlier ineffective formulations (Akhter et al., 2026). Moreover, next-generation ionizable lipids (e.g., FX12 and FC8) have demonstrated up to 100-fold improvements in editing efficiency at low doses, while enabling organ-specific targeting, such as liver (∼37%) and lung (∼16%) (Chen et al., 2025).

Additional strategies targeting endosomal escape and cell specificity further improve system performance. pH-sensitive lipids with acid-degradable linkers enable PEG shedding in endosomal environments (pH 5–6), promoting membrane destabilization and cytosolic release of RNPs. Meanwhile, receptor-specific targeting approaches—such as pseudotyping in LVNP systems—dramatically enhance selectivity, achieving up to 100% editing in ACE2+ cells and 0% in ACE2- cells, or ∼70–80% knockout in specific target populations, compared to broadly distributed but non-selective editing in conventional systems (Nielsen et al., 2024). Biological factors, including target cell activation status, also play a significant role in determining editing outcomes.

Overall, payload optimization is intrinsically linked to nanoparticle design, where synergistic interactions between cargo and carrier dictate therapeutic performance. Integration of strategies encompassing nanoparticle architecture, payload stoichiometry, formulation stability, lipid selection, and mechanisms of endosomal escape and targeting collectively drives significant improvements in CRISPR/Cas9 delivery and editing efficiency, both in vitro and in vivo. This integrated approach is essential for the development of effective, safe, and adaptable non-viral genome editing platforms, particularly for complex payloads such as base editors and prime editors, as well as for challenging disease targets including chronic viral infections.

## 5 Nanoparticle platforms for targeted delivery of CRISPR/Cas9

**Fig. 4:**
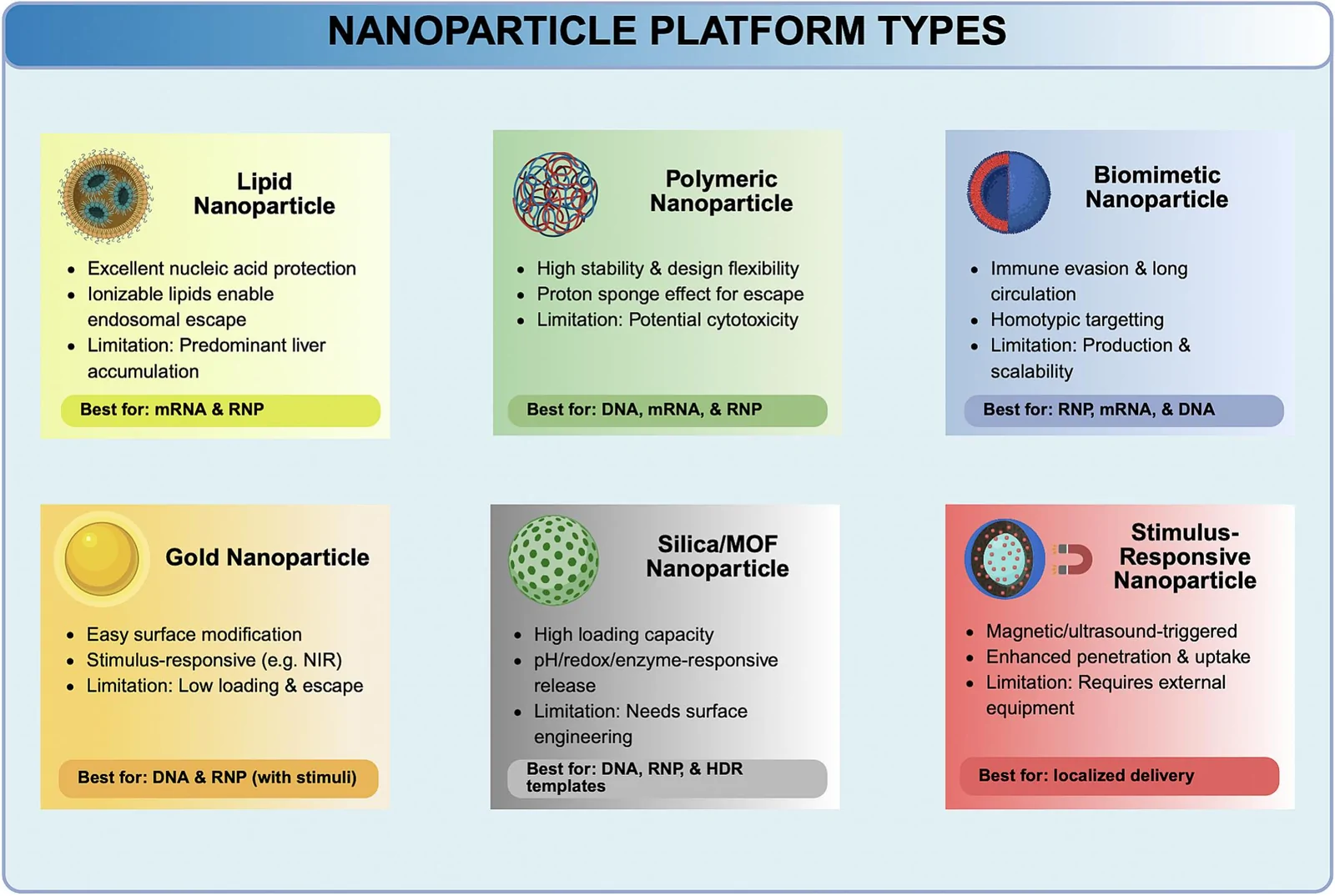
Architectural landscape, functional design principles, and applicability of major nanoparticle platforms for CRISPR/Cas9 delivery. Overview of key nanocarrier classes engineered to overcome extracellular and intracellular delivery barriers. Lipid Nanoparticles (LNPs): Provide excellent nucleic acid encapsulation and utilize ionizable lipids for endosomal disruption via protonation; primarily exhibit liver tropism and are best suited for mRNA and RNP delivery. Polymeric Nanoparticles: Offer high structural stability and chemical flexibility, facilitating endosomal escape via the proton sponge mechanism, though potential cationic cytotoxicity remains a limitation; optimal for DNA, mRNA, and RNP payloads. Biomimetic Nanoparticles: Membrane-coated carriers (e.g., cell membrane cloaking) achieve immune evasion, prolonged circulation, and homotypic cell targeting, but face manufacturing and scalability constraints; ideal for RNP, mRNA, and DNA. Gold Nanoparticles (AuNPs): Feature straightforward surface functionalization and light/NIR-responsive trigger capabilities, limited by relatively lower payload loading; best for DNA and RNP under external stimuli. Silica / Metal–Organic Framework (MOF) Nanoparticles: Characterized by high loading capacity and porous structures responsive to pH, redox, or enzymatic degradation, requiring surface engineering for stealth; optimal for DNA, RNP, and homology-directed repair (HDR) donor templates. Stimulus-Responsive Nanoparticles: Dynamically activated by external physical triggers (magnetic fields, ultrasound/cavitation) to enhance tissue penetration and localized uptake, requiring specialized external equipment; best for site-specific localized delivery. Created with BioRender.com.

Although numerous engineering strategies have been developed to overcome biological barriers in CRISPR/Cas9 delivery, their implementation is highly dependent on the nanoparticle platform employed. As summarized in Fig. 4, conventional material-based classifications are increasingly shifting toward hybrid and multifunctional systems. This transition reflects the limitation that no single material can independently address all biological barriers; thus, modern nanoparticle design emphasizes the integration of key functionalities, including circulation stability, targeted delivery, endosomal escape, and stimulus-responsive release (Tripathi et al., 2025). To provide a systematic overview, the characteristics, delivery efficiency, targeting specificity, biocompatibility, immunogenicity, and fabrication complexity of each major delivery platform are detailed in the following subsections.

### 5.1 Lipid-based nanoparticles (LNPs)

LNPs remain the most clinically advanced platform, particularly for nucleic acid and RNP-based CRISPR delivery (Marschhofer et al., 2026; Zeng et al., 2026). In terms of delivery efficiency, LNPs exhibit exceptional endocytic uptake and robust encapsulation efficiency exceeding 90%. Their primary mechanism relies on ionizable lipids (e.g., MC3, ALC-0315) that undergo protonation under acidic endosomal conditions (Huang et al., 2026), destabilizing endosomal membranes and driving cytosolic payload release to achieve in vivo knockout efficiencies of over 70% (Li et al., 2019a). Compared to submicron lipid nanoemulsions, which often suffer from physical phase instability and limited encapsulation capacity for bulky macromolecular cargoes, solid-core LNPs provide far superior structural protection and reproducible cargo loading (Pandey et al., 2022). Regarding targeting specificity, native LNPs naturally adsorb apolipoprotein E (ApoE) in circulation, leading to predominant hepatic tropism via low-density lipoprotein receptor interaction and limiting non-hepatic tissue targeting without structural re-engineering such as Selective Organ Targeting (SORT) lipid addition or ligand functionalization (Brodin et al., 2025; Kim et al., 2026; Zhang et al., 2025). From a biological safety perspective, ionizable lipids display lower acute cytotoxicity than permanent cationic lipids, yet repeated administration can trigger immune responses, including anti-PEG antibody production that induces accelerated blood clearance (ABC) and complement activation (Kim et al., 2025). Crucially, LNPs possess low fabrication complexity and high translational readiness due to standardized, highly reproducible microfluidic mixing protocols that ensure batch-to-batch consistency and scalable GMP manufacturing (Atmuri et al., 2025).

### 5.2 Polymeric nanoparticles

Polymeric nanoparticles offer high synthetic flexibility and the capacity to integrate multiple functional moieties within a single carrier. Concerning delivery efficiency and mechanisms, polymer-based systems such as PEG-PLGA and poly(beta-amino esters) (PBAE) afford controllable payload release profiles and protection against enzymatic degradation, with cationic polymers facilitating endosomal disruption via the proton sponge effect (Liu et al., 2018; Rohiwal et al., 2020; Zhu et al., 2018). However, cytosolic release efficiency varies considerably depending on molecular weight and structural architecture. Targeting specificity in basic polymeric vectors relies on passive accumulation via the enhanced permeability and retention effect, whereas active tissue targeting necessitates post-synthetic chemical conjugation of ligands, which enhances targeting precision but complicates surface charge control. A major limitation lies in biocompatibility and immunogenicity, which correlate strongly with cationic charge density; high-density cationic polymers like branched PEI induce significant cytotoxicity and membrane destabilization, whereas biodegradable formulations like PBAEs or PLGA offer superior biocompatibility (Xiu et al., 2023). To overcome this toxicity bottleneck, recent strategies utilize natural, bio-derived polysaccharide-based polymer matrices and piezoelectric biopolymers, which offer vastly superior biocompatibility and physiological responsiveness compared to synthetic cationic polymers while retaining efficient endosomal escape kinetics (Mehnath, 2025). In terms of fabrication complexity, synthetic polymers benefit from precise chemical control over functional groups, yet polydispersity management and multi-step synthesis often introduce batch-to-batch variability that poses moderate challenges for large-scale clinical scaling (Chang et al., 2026).

### 5.3 Gold nanoparticles (AuNPs)

Gold nanoparticles (AuNPs) are distinguished by their precise structural control, uniform size distribution, and versatile surface chemistry that allows facile functionalization with thiolated ligands and CRISPR payloads (Cunningham et al., 2026). Regarding delivery efficiency, metallic nanocarriers inherently suffer from low payload loading capacity and lack innate endosomal escape capabilities. To overcome these limitations, advanced AuNP platforms integrate active functional components, such as gold nanowire nanomotors or lipid–AuNP hybrid systems (LACP), which leverage external triggers like photothermal laser irradiation or ultrasound propulsion to enhance membrane penetration and achieve spatiotemporally controlled cytosolic release (Hansen-Bruhn et al., 2018; Wang et al., 2018). Furthermore, emerging stimuli-responsive nanocarrier architectures exploit localized microenvironmental triggers—such as acidic tumor pH or elevated intracellular glutathione—to induce sharp surface structural transitions, thereby enabling spatiotemporally controlled cytosolic payload release and minimizing off-target genomic cleavage (Mehnath, 2026). Targeting specificity relies heavily on passive RES accumulation or active surface functionalization with dense targeting peptides. In terms of biocompatibility and immunogenicity, spherical PEGylated AuNPs display low acute immunogenicity and minimal short-term cytotoxicity at therapeutic doses; however, concerns regarding long-term inorganic tissue retention, lack of biodegradability, and delayed off-target accumulation remain notable translational hurdles (Shi et al., 2022). Fabrication complexity is moderate, as AuNPs feature exceptional monodispersity via established reduction methods, but stoichiometric control during multi-step conjugation with complex Cas9 RNP payloads requires rigorous optimization (Ozcicek et al., 2021).

### 5.4 Silica nanoparticles and metal–organic frameworks (MOFs)

Porous inorganic carriers, including MSNs and metal–organic frameworks (MOFs, e.g., ZIF-8, ZIF-90), serve as high-capacity platforms capable of accommodating diverse and bulky CRISPR payload formats, including Cas9 RNPs and donor DNA (Xu et al., 2021). Evaluated for delivery efficiency, these platforms excel in cargo loading capacity due to their extensive surface area and tunable pore architectures. Frameworks like ZIF-8 exhibit intrinsic microenvironmental responsiveness, disassembling rapidly under acidic endosomal pH or competitive intracellular ATP binding to trigger rapid, non-denaturing cytosolic release of active RNPs (Alsaiari et al., 2018; Yang et al., 2019). Native porous particles lack intrinsic tissue targeting specificity and undergo rapid opsonization, requiring surface shielding with stealth polymers and active targeting ligands to achieve selective biodistribution (LaBauve et al., 2023; Wang et al., 2025a). Biocompatibility and immunogenicity profiles are generally favorable for biodegradable MOFs constructed with biocompatible zinc ions and organic linkers, whereas MSNs require careful tuning of particle size and pore geometry to prevent hemolysis (Fan et al., 2025; Yang et al., 2026). Regarding fabrication complexity, synthesis of MOFs and MSNs is highly reproducible under mild conditions, though achieving uniform particle size distribution during scaled-up production and complete solvent removal present key manufacturing considerations (Achenbach et al., 2025).

### 5.5 Exosomes and biomimetic nanoparticles

Biomimetic nanoplatforms, including exosomes, extracellular vesicles, and cell membrane-coated synthetic nanoparticles, represent a bio-inspired paradigm designed to overcome systemic clearance mechanisms (Wang et al., 2022; Wang et al., 2020b). In terms of delivery efficiency, biomimetic systems leverage natural membrane fusion or endocytic pathways for cellular entry; while endosomal escape efficiency in pure vesicles can be variable, hybridizing cell membranes with synthetic cores significantly enhances cytosolic payload transport. These vectors demonstrate exceptional targeting specificity, inheriting intrinsic tissue tropism from parent cell membranes (e.g., tumor homotypic targeting or macrophage inflammation homing) to enable targeted non-hepatic delivery without complex chemical functionalization (Godja and Munteanu, 2024). Their primary advantage lies in superior biocompatibility and minimal immunogenicity, as retained self-marker proteins like CD47 allow them to evade RES phagocytosis and minimize protein corona formation (Guo et al., 2024). However, fabrication complexity represents their most significant bottleneck, as isolation, purification, low loading efficiency for large Cas9 complexes, and batch-to-batch heterogeneity severely hamper standardized, large-scale clinical production compared to fully synthetic systems (Chen et al., 2022).

### 5.6 Externally stimulated and physical-assisted platforms

Externally responsive nanoplatforms—such as magnetic nanoparticles and ultrasound-responsive microbubble–nanoliposome complexes—introduce precise physical control over CRISPR delivery. Assessed for delivery efficiency, these platforms achieve rapid, high-efficiency cytosolic uptake by using external energy sources to physically propel nanoparticles or transiently permeabilize cell membranes through sonoporation, thereby bypassing classical endosomal entrapment pathways (Rohiwal et al., 2020; Ryu et al., 2020). To address the challenge of rapid systemic clearance inherent to freely circulating physical nanocarriers, recent advancements utilize hybrid delivery systems—such as nanoparticle-loaded hydrogel matrices—which provide sustained, localized delivery at the target tissue site while completely bypassing intravascular degradation and systemic off-target risks (Mehnath et al., 2023). Spatial targeting specificity is exceptionally high because payload release and cell entry occur strictly within the localized tissue region exposed to the external physical field, such as focused ultrasound or magnetic gradient fields (Ratajczak et al., 2025; Yang et al., 2024). The biocompatibility and immunogenicity of these platforms depend on their underlying material core (e.g., superparamagnetic iron oxide or lipid shells); while materials are generally well-tolerated at therapeutic doses, excessive physical energy application carries risks of localized microvascular disruption or tissue damage (Xuan et al., 2023). Finally, fabrication complexity of the nanocarriers themselves is relatively low to moderate, but overall clinical translation remains complex due to the operational requirement for specialized physical delivery devices and real-time imaging guidance during administration (Kumar et al., 2025).

## 6 Current challenges and future perspectives

### 6.1 Current challenges in targeted CRISPR/Cas9 nanoparticle delivery

While various nanoparticle platforms have demonstrated impressive efficacy in controlled preclinical models, transitioning these engineered nanocarriers from bench to bedside introduces complex translational hurdles. Beyond basic material performance, clinical viability demands strict manufacturing reproducibility, scalable production, long-term safety, and favorable regulatory profiles.

#### 6.1.1 Low intracellular delivery efficiency and endosomal escape bottlenecks

Despite significant progress, intracellular delivery efficiency remains a major bottleneck in CRISPR translation. Most nanoparticles are trapped in endosomal compartments and subsequently degraded via lysosomal pathways (Feng et al., 2024). Although various endosomal escape strategies exist, their real-world escape efficiency remains strikingly low—often yielding less than 1–2% cytosolic release—and highly inconsistent across complex in vivo environments (Dowdy, 2023; Jozić et al., 2026). This necessitates higher systemic doses, which drastically escalates acute toxicity, off-target editing risks, and systemic inflammatory responses (Feng et al., 2024). Furthermore, many delivery systems rely on a single dominant escape mechanism, such as simple pH responsiveness, without accounting for intracellular trafficking heterogeneity, resulting in variable performance across cell types (Zhu et al., 2024).

#### 6.1.2 Organ-selective delivery beyond the liver and protein corona dynamics

Targeting precision remains a paramount challenge for non-viral CRISPR therapeutics. Upon systemic administration, nanoparticle interactions with plasma proteins lead to rapid biomolecular "protein corona" formation. Opsonization by immunoglobulins and complement factors marks nanocarriers for rapid clearance by the mononuclear phagocyte system, driving non-specific accumulation in the liver and spleen (Mochalova et al., 2023; Zelepukin et al., 2024). While advantageous for hepatic applications (such as ApoE-mediated uptake via LDLRs), this intrinsic tropism severely limits effective delivery to extrahepatic tissues, such as solid tumors, the central nervous system, or hematopoietic cells. Achieving organ-selective delivery beyond the liver via SORT molecules, active ligands, or zwitterionic stealth coatings is frequently attenuated by competitive serum protein adsorption and unpredictable capillary shear stress, highlighting the difficulty of maintaining targeting fidelity in vivo (Kim et al., 2023a; Kim et al., 2023b).

#### 6.1.3 Safety, immunogenicity, anti-PEG antibodies, and repeated dosing feasibility

Safety and immunogenicity represent critical constraints, particularly when multi-dose regimes are required for complex, non-monogenic diseases. Both the bacterial Cas9 payload and synthetic nanoparticle constituents can trigger robust innate and adaptive immune responses (Stigzelius et al., 2025). Highly cationic lipids and polymers are especially prone to inducing membrane disruption, hemolysis, and systemic cytokine release (Xiu et al., 2023). Crucially, repeated administration of PEGylated nanoparticles often stimulates anti-PEG IgM/IgG production, inducing ABC and Complement Activation-Related Pseudoallergy (CARPA) that neutralize therapeutic cargoes before reaching target cells (Taghavimandi et al., 2025). Concurrently, repeat exposure can reactivate Cas9-specific neutralizing antibodies and cytotoxic T-lymphocytes. Enabling feasible multi-dosing requires transitioning toward short-lived mRNA/RNP formats, highly biodegradable ionizable lipids, and low-immunogenic zwitterionic or cell-membrane-cloaked surfaces (Wang et al., 2024).

#### 6.1.4 Limited control over intracellular trafficking pathways

Precision control over intracellular trafficking remains largely underexplored. High cellular internalization does not necessarily correlate with efficient cytoplasmic or nuclear release of CRISPR components. Different endocytic uptake mechanisms—such as clathrin-mediated endocytosis, caveolae-mediated uptake, or macropinocytosis—possess vastly different intracellular fate profiles and lysosomal degradation rates (Lee et al., 2017; Wang et al., 2016). However, most current delivery systems cannot selectively bias or direct specific internalization pathways, resulting in high variability in delivery outcomes across heterogeneous cell populations (Tang et al., 2025). This highlights the need for advanced nanocarrier designs that govern not only cellular uptake but also endosomal routing, cytosolic release timing, and nuclear translocation.

#### 6.1.5 Real-world constraints of stimuli-responsive and triggered systems

While stimuli-responsive and externally triggered nanoplatforms (e.g., pH-, redox-, light-, magnetic-, or ultrasound-responsive systems) demonstrate exceptional spatiotemporal control in vitro (Tan et al., 2026), their real-world clinical translation faces severe physical and anatomical barriers. Endogenous triggers (e.g., tumor microenvironmental pH drops or glutathione gradients) suffer from physiological heterogeneity and sluggish cleavage kinetics, leading to incomplete payload release (Lee et al., 2019). Conversely, exogenous physical triggers face tissue penetration limits (e.g., NIR light attenuation beyond a few millimeters), scattering by dense extracellular matrices, and the operational requirement for specialized, complex medical hardware during patient administration (Tran et al., 2026). Balancing trigger responsiveness with anatomical accessibility remains a pivotal constraint for these advanced platforms.

#### 6.1.6 Nanomanufacturing reproducibility, scale-up, and physical storage stability

A fundamental engineering bottleneck in translating preclinical nanocarriers lies in manufacturing scalability, lot-to-lot reproducibility, and storage shelf-life. Traditional benchtop formulation methods (such as manual mixing) rely on chaotic fluidics, yielding high batch-to-batch variability in particle size, polydispersity index (polydispersity index (PDI) < 0.1 desired), cargo loading efficiency, and surface charge (Matthew et al., 2022). Transitioning to automated microfluidic continuous-flow platforms under Good Manufacturing Practice (GMP) conditions is mandatory for scalable, reproducible assembly, though managing channel fouling and fluidic shear stress during scale-up remains challenging (Sharma et al., 2024). Furthermore, liquid formulations suffer from aggregation, lipid oxidation, and nucleic acid hydrolysis, requiring ultra-low temperature storage (−80°C) (Nomani et al., 2026). Developing robust lyophilization protocols using lyoprotectants (e.g., trehalose) is essential to ensure long-term shelf-life at ambient or refrigerated conditions (2–8°C).

#### 6.1.7 Regulatory hurdles and quality control standards

From a regulatory perspective (FDA and EMA), nanoparticle-formulated CRISPR therapies represent complex combination products situated at the intersection of gene therapy and nanomedicine (Song et al., 2024). Demonstrating safety and efficacy requires establishing rigorous Quality by Design (QbD) analytical frameworks. Major regulatory hurdles include demonstrating lot-to-lot consistency in cargo stoichiometry (Cas9-to-sgRNA ratios), validating particle size stability and PDI across manufacturing batches, quantifying free versus encapsulated payload fractions, and demonstrating complete removal of residual processing solvents (Luther et al., 2018). Furthermore, standardized high-throughput assays must be validated to quantify off-target genomic cleavage, chromosomal translocations, and long-term genotoxicity in primary human cells prior to Investigational New Drug (IND) approval (Ma et al., 2024).

### 6.2 Future perspectives in targeted CRISPR/Cas9 nanoparticle delivery

#### 6.2.1 AI-driven design of nanoparticles

Artificial intelligence and machine learning offer new opportunities for nanoparticle design. These data-driven approaches enable prediction of structure–function relationships and rapid optimization of physicochemical parameters. Integration with multi-omics data may facilitate identification of materials tailored to specific biological contexts, reducing reliance on trial-and-error strategies (Han et al., 2025).

#### 6.2.2 Personalized and precision delivery systems

Personalized medicine is increasingly applied to delivery system design. This approach considers individual genetic variation, tissue microenvironment, and disease-specific conditions. Strategies include ligand-targeted nanoparticles, stimulus-responsive systems, and adaptive materials that respond to biological environments, aiming to enhance specificity while minimizing off-target effects (Imani et al., 2025).

#### 6.2.3 Integration with next-generation CRISPR systems

Emerging technologies such as base editing and prime editing offer higher precision and reduced reliance on DSBs. Integration with nanoparticle delivery systems is expected to significantly improve safety and efficiency. Alternative CRISPR systems, including Cas12 and Cas13, further expand therapeutic targets to RNA (Ali Agha et al., 2025; Hillary and Ceasar, 2023).

#### 6.2.4 Multi-functional and hybrid nanoplatforms

Future approaches emphasize multifunctional platforms capable of overcoming multiple barriers simultaneously. Hybrid systems combining lipids, polymers, and peptides integrate targeting, endosomal escape, and controlled release. Biomimetic approaches, such as exosome-based systems, further enhance biocompatibility and exploit natural transport pathways (Godja and Munteanu, 2024; Rajana et al., 2022).

#### 6.2.5 Toward clinical translation

**Fig. 5:**
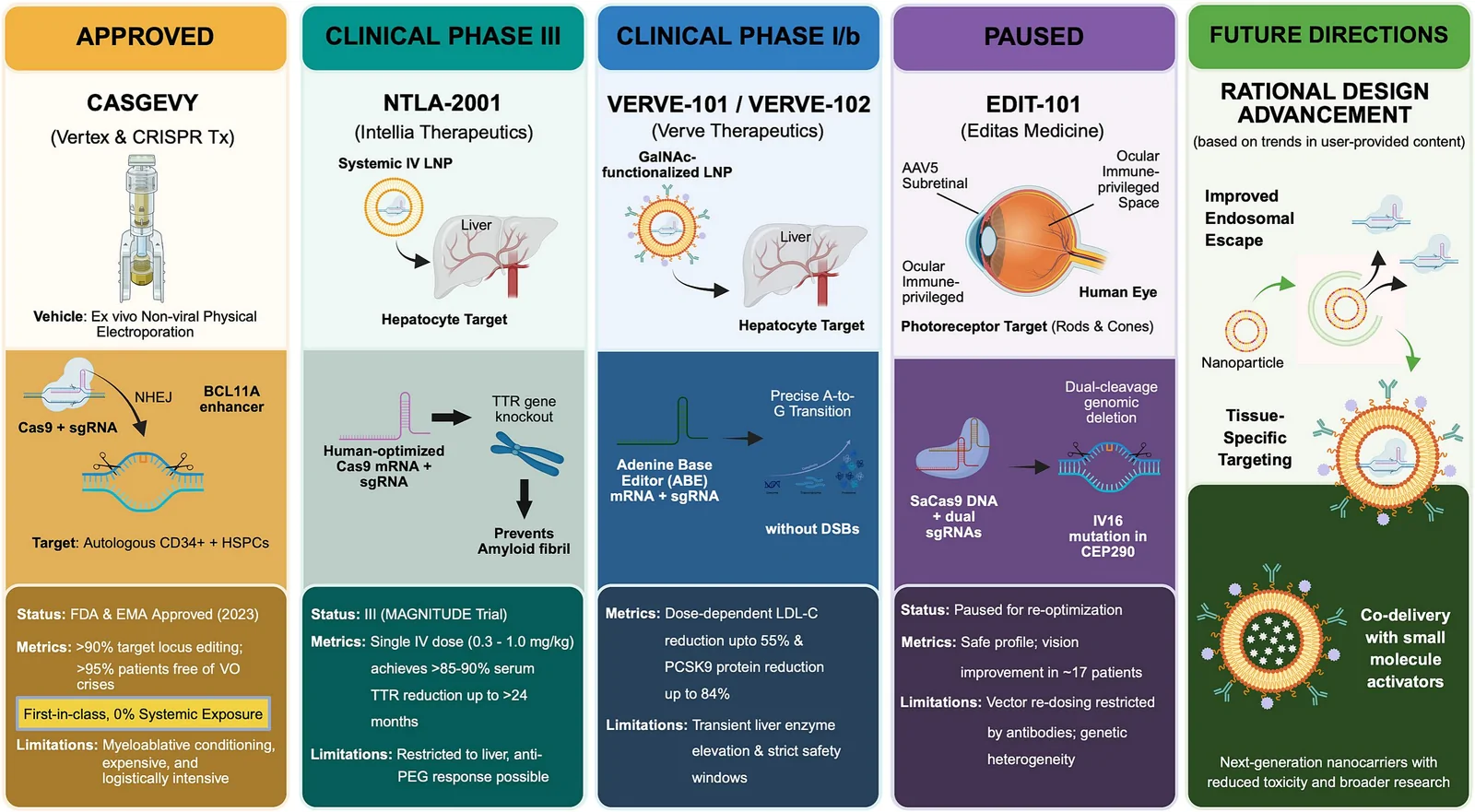
Approved and clinical-stage landscape of CRISPR/Cas9 delivery systems. Schematic overview illustrating the clinical translation of genome editing platforms: (1) Approved Platform (CASGEVY): Ex vivo electroporation of autologous CD34^+^ HSPCs delivering Cas9 mRNA/sgRNA for BCL11A enhancer disruption to treat SCD and TDT (>90% editing; zero systemic vehicle exposure); (2) Phase III (NTLA-2001): Systemic IV LNP (pKa 6.1) utilizing ApoE corona targeting LDLR for TTR gene knockout (>85–90% TTR reduction up to 24+ months; constrained by liver tropism); (3) Phase I/b (VERVE-101/102): GalNAc-functionalized LNPs targeting ASGPR receptors delivering ABE mRNA for PCSK9 base editing without DSBs (55% LDL-C reduction); (4) Paused Platform (EDIT-101): Subretinal AAV5 delivery of SaCas9 for CEP290 gene excision (paused due to vector immunogenicity and re-dosing limits); and (5) Future Directions: Next-generation nanocarriers optimized for extrahepatic targeting, enhanced endosomal escape, and reduced toxicity. Created with BioRender.com.

The clinical translation of CRISPR/Cas9 genome editing therapies depends not only on biological performance but also on manufacturability, scalability, safety margins, and predictable in vivo biodistribution. While preclinical studies have yielded vast arrays of functionalized nanocarriers, bridging the gap to human application requires rigorous optimization of delivery safety, tissue tropism, and transient activity profiles. To illustrate how nanoparticle design principles dictate clinical success, landmark clinical-stage and FDA-approved CRISPR delivery platforms—spanning both ex vivo and in vivo modalities—demonstrate distinct translational trajectories from bench to bedside. As detailed in Fig. 5, these systems highlight how carrier architecture, ligand modification, and payload choice directly influence clinical efficacy, tissue-specific tropism, and regulatory feasibility.

Among these platforms, ex vivo delivery systems such as Casgevy (exagamglogene autotemcel) represent the most clinically mature paradigm, achieving full regulatory approval by utilizing physical electroporation to transduce autologous CD34+ hematopoietic stem cells (Lesmana et al., 2024). This approach circumvents systemic exposure and protein corona interference, ensuring high editing efficiency without off-target organ uptake, though its broad application remains constrained by complex manufacturing logistics and the requirement for myeloablative conditioning (Hoy, 2024). Conversely, systemic in vivo delivery platforms have achieved remarkable breakthroughs by exploiting endogenous biological mechanisms. A prime example is NTLA-2001, an intravenous LNP formulation that relies on plasma ApoE adsorption to form a biomolecular corona, directing targeted uptake by low-density lipoprotein receptors (LDLR) on hepatocytes (Li et al., 2025b). By employing a pH-sensitive ionizable lipid, NTLA-2001 facilitates robust endosomal escape and delivers sustained target gene knockdown in human patients following a single infusion (Haghighi et al., 2024).

To further refine tissue tropism and safety, second-generation clinical candidates have advanced toward active ligand-mediated targeting and non-cleaving editing mechanisms. Platforms such as VERVE-101 and its successor VERVE-102 utilize GalNAc-functionalized LNPs to directly engage asialoglycoprotein receptors (ASGPR) on hepatocytes, enabling precise delivery of mRNA encoding adenine base editors (Flight et al., 2025; Vafai et al., 2024). This active targeting strategy, combined with base editing that avoids double-strand DNA breaks, mitigates the risks of chromosomal translocations and reduces off-target cytotoxicity (Lee et al., 2023). In contrast, viral-based local delivery systems such as EDIT-101 have demonstrated the feasibility of subretinal AAV delivery for ocular diseases; however, clinical challenges regarding neutralizing immunity, limited vector re-dosing, and inter-patient genetic variability have shifted significant momentum toward non-viral LNP alternatives (Collin and Leroy, 2024; Pierce et al., 2024).

Moving these nanoparticle-based CRISPR therapies from preclinical models to clinical registration requires overcoming critical translational bottlenecks. At the preclinical formulation stage, precise tuning of ionizable lipid protonation profiles (pKa≈6.0–6.5) and cargo selection—favoring transient mRNA or RNP complexes over pDNA—remains essential for minimizing systemic toxicity and off-target cleavage (Im et al., 2024). Furthermore, translating these systems requires navigating species-specific protein corona dynamics, as mouse plasma binding profiles often fail to predict human biodistribution, necessitating validation in non-human primate models (Tay et al., 2020). On the manufacturing front, benchtop formulation methods must transition to automated microfluidic production technologies. Microfluidic processes enable precise control over nanoparticle size, composition, polydispersity, and batch-to-batch reproducibility, which are critical for meeting stringent GMP and regulatory compliance standards (Seder et al., 2024).

Additionally, the ability to support repeat dosing without reduced efficacy or accelerated blood clearance triggered by anti-PEG antibodies is essential for expanding non-hepatic therapeutic indications. Developing stable, safe, and scalable nanomanufacturing systems therefore remains a top priority. Ultimately, no single engineering strategy can independently overcome all delivery barriers. Future success will depend on integrating advanced material design, precise active targeting, and next-generation genome editing technologies. Endosomal escape remains the primary bottleneck, and continued innovation in this area will be crucial for achieving safe, robust, and broadly accessible clinical applications (Chatterjee et al., 2024).

## 7 Conclusion

CRISPR/Cas9 therapeutics offer significant potential for treating genetic diseases, yet their clinical translation is limited by complex biological barriers across extracellular, cellular, and intracellular levels. This review emphasizes that delivery efficiency is governed by interconnected processes, with endosomal escape remaining a major bottleneck. Advances in nanoparticle engineering—particularly ligand-mediated targeting, PEGylation, biomimetic coatings, and stimulus-responsive systems—have substantially improved delivery performance. The emergence of multifunctional and hybrid nanoplatforms enables simultaneous overcoming of multiple barriers, marking a shift toward integrated design strategies. In parallel, optimization of CRISPR payloads, including format, stability, and carrier compatibility, plays a critical role in enhancing editing efficiency and safety. Despite progress, challenges such as limited targeting precision, intracellular variability, and immunogenicity persist. Future developments integrating artificial intelligence, precision delivery, and next-generation CRISPR systems are expected to accelerate the development of safe and effective genome editing therapies.

## CRediT authorship contribution statement

**Sriwidodo Sriwidodo:** Writing – review & editing, Writing – original draft, Conceptualization. **Cecep Suhandi:** Writing – review & editing, Supervision, Methodology. **Salsa Sagitasa:** Writing – review & editing, Visualization, Software. **Belva Annora Alfita Davinali:** Writing – review & editing, Visualization, Software. **Iman Permana Maksum:** Writing – review & editing, Validation, Data curation. **Sabreena Safuan:** Writing – review & editing, Supervision, Data curation.

## Declaration of generative AI and AI-assisted technologies in the writing process

During the preparation of this work, the author(s) used ChatGPT (OpenAI) to assist in proofreading and improving the readability of the initial manuscript draft. After using this tool, the author(s) carefully reviewed and edited the content as needed and take full responsibility for the content of the published article.

## Declaration of competing interest

The authors declare that they have no known competing financial interests or personal relationships that could have appeared to influence the work reported in this paper.

## Data Availability

No data was used for the research described in the article.
